# Multifunctional Fluorescent Probe for Simultaneous Detection of ATP, Cys, Hcy, and GSH: Advancing Insights into Epilepsy and Liver Injury

**DOI:** 10.1002/advs.202415882

**Published:** 2025-01-30

**Authors:** Ting Yu, Yang Li, Jing Li, Yabing Gan, Zhengze Long, Yun Deng, Youyu Zhang, Haitao Li, Peng Yin, Shouzhuo Yao

**Affiliations:** ^1^ Institute of Interdisciplinary Studies Hunan Normal University Changsha 410081 China; ^2^ Key Laboratory of Chemical Biology and Traditional Chinese Medicine Research (Ministry of Education) College of Chemistry and Chemical Engineering Hunan Normal University Changsha 410081 China; ^3^ College of Life Sciences Hunan Normal University Changsha 410081 China

**Keywords:** adenosine triphosphate, biothiols, fluorescent probe, simultaneous sensing

## Abstract

Adenosine triphosphate (ATP) is a critical intracellular energy currency that plays a key role in various cellular processes and is closely associated with numerous diseases. Similarly, biothiols such as glutathione (GSH), cysteine (Cys), and homocysteine (Hcy) are integral to many physiological and pathological processes due to their strong redox properties. Simultaneous discrimination and detection of ATP and biothiols offer valuable insights into the pathogenesis of conditions such as epilepsy and liver injury. This study introduces the first fluorescent probe, **BCR**, designed for multifunctional detection of ATP, GSH, Hcy, and Cys. With outstanding optical properties, excellent biocompatibility, high selectivity, and superior sensitivity, probe **BCR** enables effective imaging of ATP and biothiol dynamics in vivo. Moreover, probe **BCR** successfully visualizes changes in ATP, GSH, Hcy, and Cys levels in a PTZ‐induced epileptic zebrafish model and an APAP‐induced mouse liver injury tissue section model. These findings underscore the significant potential of probe **BCR** for early disease diagnosis and therapeutic applications.

## Graphical abstract

A multifunctional fluorescent probe, **BCR**, is designed for the simultaneous detection of adenosine triphosphate (ATP), gluotathione (GSH), homocysteine (Hcy), and cysteine (Cys). Furthermore, probe **BCR** successfully visualizes changes in ATP, GSH, Hcy, and Cys levels in a PTZ‐induced epileptic zebrafish model and an APAP‐induced mouse liver injury tissue section model.

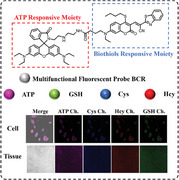

## 1 Introduction

Adenosine triphosphate (ATP), composed of adenine, ribose, and three phosphate groups, is a fundamental intracellular energy storage molecule in biological organisms.^[^ ^1^, ^2^, ^3^ ^]^ It plays a central role in regulating cellular metabolic activities, energy transfer, enzymatic catalysis, biosynthesis, and other critical cellular functions.^[^ ^4^, ^5^, ^6^ ^]^ Additionally, ATP is involved in neurotransmission, ion channel activity, and the synthesis of genes and proteins.^[^ ^7^, ^8^, ^9^ ^]^ Abnormal ATP concentrations are linked to various neurodegenerative diseases, including epilepsy, Parkinson's disease, cardiovascular disorders, and Alzheimer's disease.^[^ ^2^, ^10^ ^]^ These conditions are often associated with abnormal oxidative stress and disruptions in energy metabolism.^[^ ^11^, ^12^ ^]^ Glutathione (GSH), an endogenous antioxidant, is crucial for maintaining redox balance and regulating metabolism within the intracellular microenvironment.^[^ ^13^ ^]^ It protects thiol‐containing proteins from oxidative damage, alleviates liver injury, and enhances the antioxidant capacity of cancer patients.^[^ ^14^, ^15^ ^]^ Homocysteine (Hcy) and cysteine (Cys), which share structural and functional similarities, also play vital roles in biological systems.^[^ ^16^ ^]^ While Hcy serves as a precursor for Cys biosynthesis, it performs unique physiological functions.^[^ ^17^ ^]^ Hcy is known to increase reactive oxygen species (ROS) and induce oxidative damage in cells, whereas Cys exhibits antioxidant properties.^[^ ^18^ ^]^ Alterations in the levels of these biothiols are associated with a spectrum of conditions, including neurodegenerative disorders, cardiovascular and cerebrovascular diseases, and liver injury.^[^ ^19^, ^20^, ^21^ ^]^ Given the interlinked roles of ATP and biothiols in cellular homeostasis and disease progression, the ability to simultaneously monitor their levels is of significant value.^[^ ^4^, ^22^ ^]^ This capability could provide deeper insights into the pathophysiological mechanisms underlying neurological and hepatic diseases and facilitate the development of targeted diagnostic and therapeutic approaches.^[^ ^23^ ^]^

Traditional methods for the detection of biothiols and ATP, such as High‐Performance Liquid Chromatography (HPLC), Mass Spectrometry (MS), Electrochemical Methods (EM), and Atomic Absorption Spectrometry (AAS), have been extensively reported.^[^ ^24^, ^25^ ^]^ However, these techniques often face limitations such as low sensitivity, cumbersome sample preparation, and the need for complex instrumentation, making them less suitable for biological applications.^[^ ^26^, ^27^, ^28^ ^]^ In contrast, fluorescent probe technology has gained widespread use for detecting biothiols and ATP in complex biological systems, owing to its advantages of in situ detection, high sensitivity, low cost, and strong selectivity.^[^ ^29^, ^30^, ^31^ ^]^ To date, numerous fluorescent probes have been developed for detecting ATP and other analytes.^[^ ^4^, ^30^, ^31^, ^32^ ^]^ Dual functional fluorescent probes for simultaneous monitoring ATP and various biological species, such as hydrogen peroxide (H₂O₂),^[^ ^23^, ^32^ ^]^ hydrogen sulfide (H₂S),^[^ ^30^, ^33^ ^]^ Glutathione (GSH),^[^ ^34^ ^]^ superoxide anion (O₂^•⁻^),^[^ ^35^ ^]^ peroxynitrous acid (ONOO⁻),^[^ ^36^, ^37^ ^]^ and ferrous ion (Fe^2+^),^[^ ^22^, ^38^ ^]^ have been extensively studied (Table S1, Supporting Information).^[^ ^35^, ^39^ ^]^ However, fluorescent probes capable of simultaneously detecting ATP, Cys, Hcy, and GSH remain scarce. For example, in 2021, Chai et al.^[^ ^34^ ^]^ developed a DNA aptamer probe (A‐G/NT) that leveraged nanoparticle assembly to achieve spatially controlled and gated imaging of ATP and glutathione (GSH) within mitochondria.^[^ ^30^ ^]^ This system enabled multiplexed imaging of mitochondrial GSH and ATP in tumor‐bearing mice. More recently, in 2024, Lang's groups^[^ ^7^ ^]^ successfully developed a bifunctional coumarin‐linked tetraphenylethylene (TPE) probe, designated **P1**. This probe localizes to mitochondria and allows visualization of ATP and GSH in living cells and zebrafish. Despite these advancements, existing probes are unable to differentially detect ATP, GSH, cysteine (Cys), and homocysteine (Hcy), as these biothiols often exhibit mutual interference.^[^ ^40^ ^]^ This limitation underscores the urgent need to develop fluorescent probes capable of simultaneously distinguishing and detecting GSH, Hcy, Cys, and ATP in multiple detection channels.^[^ ^41^ ^]^ Such advancements could significantly enhance the precision of biochemical monitoring in diverse biological and pathological contexts.

**Scheme 1:**
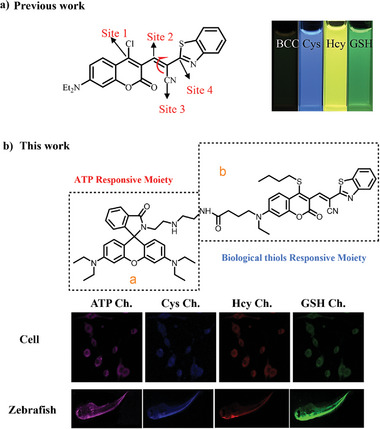
a) Previous work for the simultaneous sensing of Cys, Hcy, and GSH, b) Structure of probe **BCR** and its application. [Correction added on 10 February 2025, after first online publication: Scheme 1 image updated.]

Based on our previous research and other studies,^[^ ^4^, ^13^, ^17^ ^]^ we have rationally designed and synthesized a multifunctional fluorescent probe, **BCR**, to enable the simultaneous detection and fluorescence imaging of GSH, Hcy, Cys, and ATP (**Scheme** **1**). The design incorporates a piperazine ring as a central bridge, linking coumarin and rhodamine, creating two reactive sites that independently target biothiols and ATP without mutual interference. The coumarin derivative in the probe includes multiple binding sites that facilitate highly selective recognition of Cys, GSH, and Hcy. Meanwhile, the rhodamine derivative, with its multiple amino groups, efficiently and specifically captures ATP. This molecular optimization strategy ensures rapid and simultaneous identification of GSH, Hcy, Cys, and ATP in living systems. Furthermore, this probe has been successfully applied to the simultaneous discrimination of endogenous GSH, Hcy, Cys, and ATP in cells and zebrafish. It has also been utilized to investigate changes in the levels of GSH, Hcy, Cys, and ATP in animal models of epilepsy and acute liver injury. These findings underscore the potential of probe **BCR** for real‐time monitoring and research in complex biological systems.

## 2 Results and Discussion

### 2.1 Design and Synthesis of Probe BCR

**Scheme 2:**
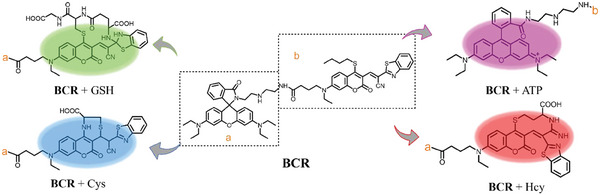
Proposed sensing mechanisms of probe **BCR** for ATP and biothiols.

As illustrated in **Scheme** **2**, the multifunctional fluorescent probe **BCR** was successfully designed, synthesized, and comprehensively characterized using NMR, IR, and HRMS (Figures S26–S29, Supporting Information). In the molecular design, two critical considerations were prioritized. First, the probe's responsive groups for ATP and thiols (mercaptans) were selected to ensure high sensitivity and specificity in complex living systems. Second, the fluorescence emission peaks of the fluorophores for ATP and thiols were carefully engineered to be well‐separated, minimizing spectral crosstalk. This design enables the simultaneous, reliable imaging of ATP and biologically relevant thiols in living organisms. To meet these criteria, and inspired by our previous work, we selected a piperazine ring as the central molecular scaffold. This structure effectively bridges the functional fluorophores while maintaining flexibility and stability. Rhodamine B and coumarin derivatives were chosen as the fluorophores, tailored for ATP and thiol recognition, respectively. Specifically, rhodamine spironolactone derivatives were incorporated, as their multiple amino groups render them highly sensitive and selective toward ATP.^[^ ^32^ ^]^ Coumarin derivatives, on the other hand, possess multiple binding sites, allowing simultaneous and selective recognition of GSH, Cys, and Hcy. By combining these components, we constructed a multifunctional fluorescent probe, **BCR**, capable of selectively and sensitively detecting ATP and biothiols. This innovative design provides a robust tool for monitoring these critical analytes in living systems, facilitating the study of their dynamic roles in various biological processes.

### 2.2 Spectral Response of Probe BCR

**Figure 1:**
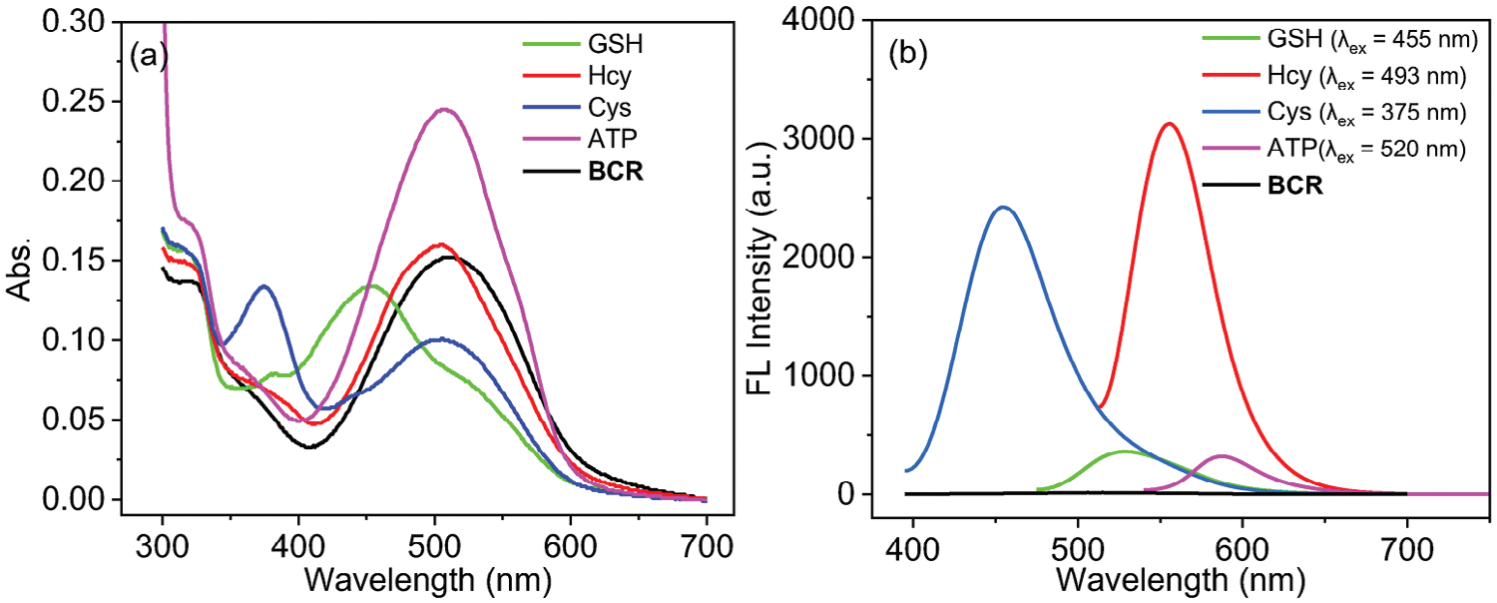
a) Absorption spectra, b) fluorescence spectra of probe **BCR** (10 µm) upon addition of GSH/Hcy/Cys (100 µm), ATP (10 mm) at room temperature for 30 min.

Initially, we optimized the spectral conditions for the interactions of probe **BCR** with biological thiols and ATP (Figures S1–S5, Supporting Information). Under these optimized conditions, the addition of GSH, Hcy, and Cys to probe **BCR** led to significant fluorescence intensity enhancements at distinct wavelengths: 529 nm (green channel, excitation at 455 nm), 555 nm (red channel, excitation at 493 nm), and 455 nm (blue channel, excitation at 375 nm), respectively. Furthermore, in the presence of ATP, a pronounced enhancement of purple fluorescence was observed at 587 nm (excitation at 520 nm) (**Figure** **1**a,b). These results demonstrate that probe**BCR** can effectively differentiate GSH, Hcy, Cys, and ATP based on their specific excitation and emission wavelengths, enabling simultaneous multi‐analyte detection. The high sensitivity and specificity of **BCR** toward these analytes underscore its potential as a robust tool for multi‐target detection in complex biological systems and environmental monitoring. This capability highlights its suitability for applications requiring real‐time, in situ analysis, such as tracking dynamic biochemical changes and assessing metabolic states.

**Figure 2:**
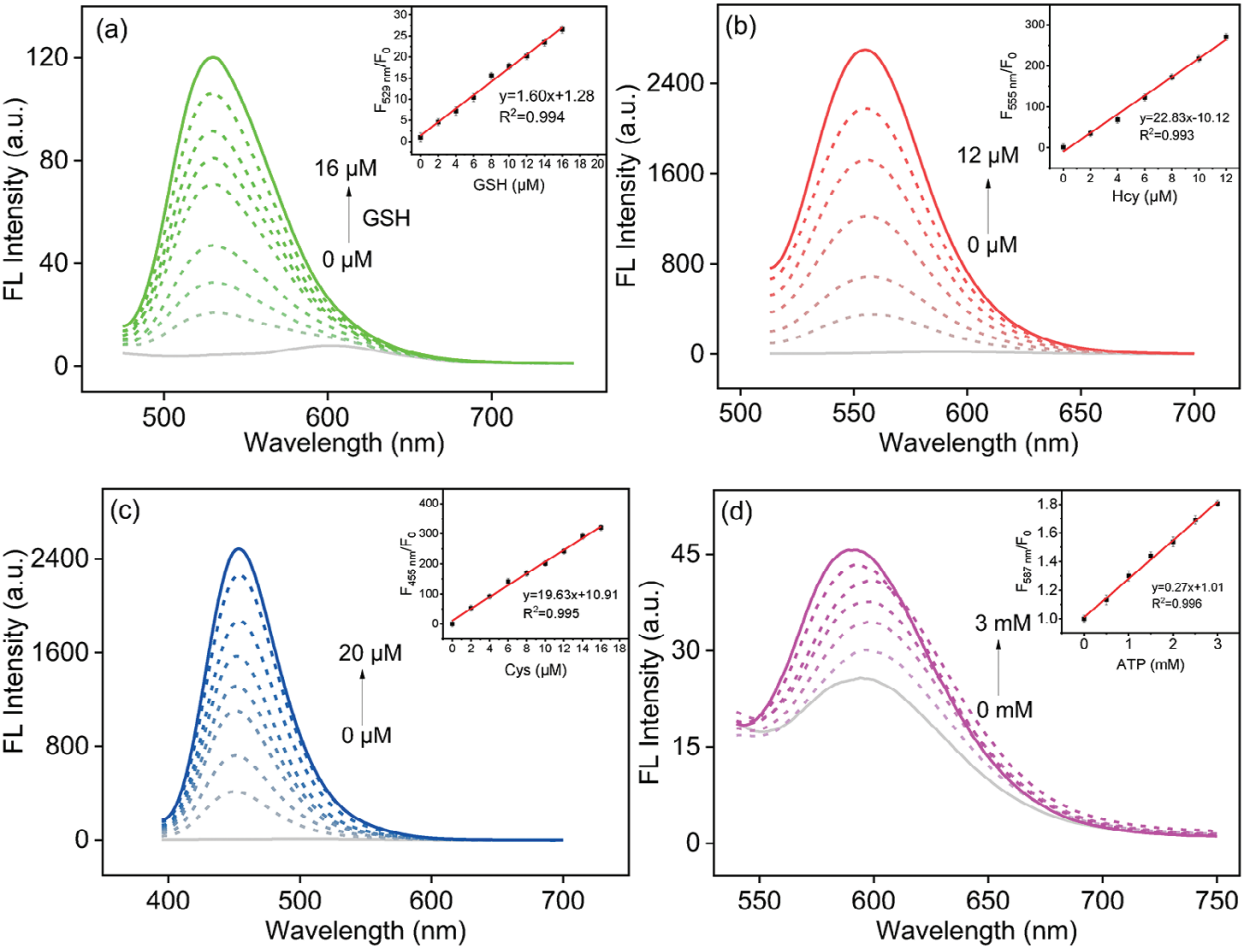
Fluorescence spectra of probe **BCR** solution after adding different concentrations of c) GSH (0–16 µm), d) Hcy (0–12 µm), e) Cys (0–20 µm) and f) ATP (0–3 mm). Conditions: λ_ex_ = 455 nm for GSH, λ_ex_ = 493 nm for Hcy, λ_ex_ = 375 nm for Cys, λ_ex_ = 520 nm for ATP, slit (nm): GSH: 2.5/5; Hcy: 2.5/5; Cys: 5/5; ATP: 2.5/5.

In addition, the fluorescence intensity of probe **BCR** increased progressively with rising concentrations of GSH, Hcy, and Cys. Notably, the fluorescence intensity exhibited a strong linear correlation with GSH concentrations ranging from 0 to 16 µm at 529 nm (green channel). Similarly, linear relationships were observed for Hcy (0–12 µm) at 555 nm (red channel) and for Cys (0–20 µm) at 455 nm (blue channel). Concurrently, as the ATP concentration increased from 0 to 3 mm, the fluorescence intensity at 587 nm (purple channel) rose significantly, displaying a well‐defined linear relationship. The limits of detection (LOD) for Cys, Hcy, GSH, and ATP, calculated based on S/N = 3, were determined to be 12.5 nm, 1.0 nm, 2.4 nm, and 31.8 µm, respectively (**Figure** **2**). These values demonstrate the high sensitivity of probe **BCR** across multiple analytes. The distinct emission bands for each target further validate the probe's capability for simultaneous multi‐analyte detection. Collectively, these findings highlight the exceptional sensitivity and specificity of probe **BCR** in detecting biological thiols and ATP. Its ability to operate across multiple emission channels underscores its potential as a versatile and practical tool for real‐time biochemical analysis in complex biological and environmental systems.

Selectivity is a crucial criterion for evaluating the performance of fluorescent probes. To assess the selectivity of probe **BCR** toward biological thiols and ATP, various analytes were tested. As shown in Figure S6 (Supporting Information), GSH, Cys, Hcy, and ATP induced significant fluorescence enhancements in their respective channels. In contrast, the presence of competing species caused negligible fluorescence changes, demonstrating that **BCR** exhibits excellent selectivity for GSH, Cys, Hcy, and ATP even in complex biological environments.

Next, we evaluated the anti‐interference performance of **BCR** recognizing ATP, GSH, Cys, and Hcy. GSH, Cys, Hcy, and ATP were sequentially added to the **BCR** solution containing various analytes (100 µm). The fluorescence intensity of **BCR** (Figure S7, Supporting Information) was significantly increased, demonstrating that the specific recognition of Cys, Hcy, and ATP by **BCR** was not affected by the presence of other biological species, such as reactive oxygen species, active sulfur species, cations, anions, amino acids.

The time‐dependent fluorescence responses of probe **BCR** to biothiols and ATP were also investigated. As shown in Figures S8–S11 (Supporting Information), upon the addition of 100 µm GSH, Hcy, or Cys to **BCR**, fluorescence intensities reached equilibrium at 18, 20, and 13 min, respectively. Similarly, ATP (100 µm) produced fluorescence enhancement that stabilized within 23 min. These results confirm that **BCR** is capable of rapid and reliable detection of its target analytes under biologically relevant conditions.

In addition, the photostability of the probe **BCR** and the time‐dependent stability of the fluorescence intensity during its reaction with the analyte were evaluated. As shown in Figure S12 (Supporting Information), the fluorescence of probe **BCR** and its reaction products remained stable for at least 20 min, highlighting its suitability for real‐time imaging applications.

To evaluate the ability of **BCR** to differentiate between ATP and biothiols in complex environments, the effect of pH on the fluorescence behavior of **BCR** was investigated in the absence and presence of ATP and biothiols across different pH levels. As shown in Figure S13 (Supporting Information), **BCR** exhibited negligible fluorescence within the pH range of 3.0–6.0. However, upon treatment with each biothiol, the probe reacted with GSH, Hcy, Cys, and ATP at pH 6.0–9.0, resulting in significant fluorescence enhancement. These results demonstrate that **BCR** effectively differentiates GSH, Hcy, Cys, and ATP under physiological conditions, making it suitable for further biological application.

### 2.3 Fluorescence Imaging of GSH, Hcy, Cys, and ATP Dynamics in Living Cells During Epileptic Events

Inspired by the excellent spectral characteristics of GSH, Hcy, Cys, and ATP demonstrated by probe **BCR**, we explored its imaging capability to simultaneously detect these analytes in living cells. To evaluate the suitability of probe **BCR** for biological applications, its biocompatibility was assessed via an MTT (3‐(4,5‐dimethylthiazol‐2‐yl)‐2,5‐diphenyltetrazolium bromide) assay. When cells were incubated with varying concentrations of probe **BCR** for 24 h, the survival rate exceeded 90% (Figure S14, Supporting Information). This result indicates that probe **BCR** exhibits low cytotoxicity, and good biocompatibility, and is well‐suited for bioimaging applications, providing a robust foundation for further experiments.

We next investigated the ability of probe **BCR** to respond to GSH, Hcy, Cys, and ATP in SH‐SY5Y and HepG2 cells. As shown in Figure S15 (Supporting Information), both cell lines exhibited strong fluorescence signals in the specific emission channels for GSH (green), Hcy (red), Cys (blue), and ATP (purple) after incubation with probe **BCR** (5 µm) for 45 min. To validate specificity, cells were pre‐treated with N‐ethylmaleimide (NEM, a biothiol scavenger) or oligomycin A (Omy A, an ATP scavenger) before probe incubation. The respective biothiol and ATP channels showed almost no fluorescence, confirming selective quenching.

Subsequently, cells were pre‐treated with NEM or Omy A, followed by incubation with exogenous GSH, Hcy, Cys, or ATP along with probe **BCR**. This resulted in the reappearance of strong, specific fluorescence in the green, red, blue, and purple channels, respectively. Endogenous and exogenous fluorescence imaging in HeLa cells produced similar results, confirming the probe's utility across multiple cell types. These findings demonstrate that probe **BCR** is a powerful tool for real‐time visualization and monitoring of changes in endogenous and exogenous levels of GSH, Hcy, Cys, and ATP in living cells.

**Figure 3:**
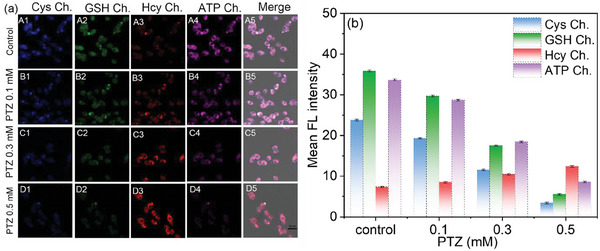
a) Confocal fluorescence imaging of GSH, Hcy, Cys, and ATP in SH‐SY5Y cells. Cells were incubated with a probe (5 µm) for 30 min (A1–A5). The cells were pretreated with PTZ (0.1 mmol l^−1^) (B1‐B5), PTZ (0.3 mmol l^−1^) (C1–C5) and PTZ (0.5 mmol l^−1^) (D1‐D5) for 12 h, and incubated with probe (5 µm) for 30 min. b) Relative pixel intensity of the fluorescence images A1–D5 in (a). *λ*_ex_ = 405 nm, *λ*_em_ = 420–470 nm for the blue channel, *λ*_ex_ = 458 nm, *λ*_em_ = 500–560 nm for the green channel, and *λ*_ex_ = 488 nm, *λ*_em_ = 540–630 nm for the red channel, and *λ*_ex_ = 514 nm, *λ*_em_ = 580–650 nm for the purple channel. Scale: 25 µm.

Epilepsy, a common chronic neurological disorder, is closely associated with oxidative stress.^[^ ^42^ ^]^ To further explore the utility of probe **BCR**, we constructed an SH‐SY5Y cell injury model induced by pentylenetetrazol (PTZ), a GABAA antagonist commonly used to establish epilepsy models. We monitored the levels of GSH, Hcy, Cys, and ATP in this model (**Figure** **3**). SH‐SY5Y cells were pre‐treated with increasing concentrations of PTZ (0, 0.1, 0.3, and 0.5 mm) for 12 h, followed by incubation with probe **BCR** (5 µm) for 30 min. As shown in Figure 3, fluorescence intensities for GSH, Cys, and ATP gradually decreased with higher PTZ concentrations, indicating a reduction in their intracellular levels. Conversely, the fluorescence signal in the Hcy channel increased, likely due to PTZ‐induced autooxidation of Hcy, resulting in the formation of reactive oxygen species (ROS). The decrease in GSH and Cys levels can be attributed to their roles as endogenous antioxidants, maintaining the cellular redox balance under oxidative stress. ATP, a marker of energy metabolism, is often secreted into the extracellular environment under oxidative stress conditions to preserve cellular homeostasis. These results suggest that probe **BCR** effectively captures the dynamic changes in biothiol and ATP levels during oxidative stress, providing valuable insights into the molecular mechanisms of epilepsy progression and oxidative stress‐induced damage.

### 2.4 Real‐Time Fluorescence Imaging of GSH, Hcy, Cys, and ATP Dynamics in Zebrafish During Epileptic Events

To further evaluate the biological application of probe **BCR**, we conducted in vivo experiments using zebrafish. As shown in Figure S16 (Supporting Information), zebrafish pre‐treated with N‐ethylmaleimide (NEM) and subsequently incubated with probe **BCR** displayed almost no fluorescence signal during imaging, indicating successful quenching of biothiol‐associated channels. Upon the addition of exogenous GSH, Hcy, and Cys, distinct fluorescence responses appeared in their respective channels, confirming the probe's ability to selectively detect these analytes in a living organism. Similarly, ATP imaging yielded comparable results. Zebrafish pre‐treated with oligomycin A (Omy A, an ATP scavenger) and then incubated with probe **BCR** exhibited negligible fluorescence in the ATP‐specific channel. However, when exogenous ATP was introduced, fluorescence reappeared in the corresponding ATP emission channel. These findings, consistent with the results of cellular imaging experiments, demonstrate that probe **BCR** can effectively distinguish GSH, Hcy, Cys, and ATP in vivo through multicolor fluorescence imaging.

**Figure 4:**
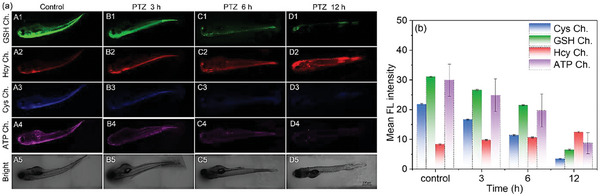
a) Confocal fluorescence imaging of GSH, Hcy, Cys, and ATP in Zebrafish. Probe **BCR** (5 µm) was incubated with zebrafish for 45 min (A1–A5), and added (6 mm) PTZ, and incubated with zebrafish for 3 h (B1–B5), 6 h (C1–C5), and 12 h (D1–D5), respectively. b) The relative pixel intensity of the images in different groups corresponding to (a). Scale: 200 µm.

We selected zebrafish as the animal model due to their high genetic homology with humans, rapid development, and ease of handling.^[^ ^43^ ^]^ As shown in **Figure** **4**, zebrafish incubated with probe **BCR** (5 µm) exhibited fluorescence signals across all four channels, corresponding to GSH, Hcy, Cys, and ATP. In the PTZ‐induced epilepsy model, the fluorescence intensity in the GSH, Cys, and ATP channels progressively weakened with increased incubation time, while fluorescence intensity in the Hcy channel significantly increased. These trends mirror the results observed in cell imaging experiments, further confirming that oxidative stress and redox imbalances occur during epileptic episodes. These results indicate that probe **BCR** is a powerful tool for real‐time, multicolor fluorescence imaging of bio‐thiol and ATP dynamics in vivo. Its application in zebrafish highlights its potential for studying biochemical changes during epilepsy and other oxidative stress‐related conditions, providing valuable insights into the underlying mechanisms.

### 2.5 Biothiol and ATP Dynamics in Cells and Mouse Tissues Within a DILI Model

Studies have demonstrated that acetaminophen (APAP) is widely used for its analgesic and antipyretic properties.^[^ ^44^, ^45^ ^]^ However, during its metabolism, APAP can induce liver damage by generating excessive reactive oxygen species (ROS) and triggering oxidative stress.^[^ ^46^, ^47^ ^]^ To evaluate whether probe **BCR** can assess liver injury through changes in GSH, Hcy, Cys, and ATP levels, we established an APAP‐induced liver injury (DILI) model using HepG2 cells.

**Figure 5:**
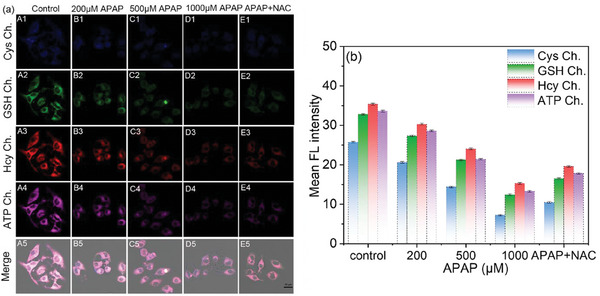
a) Confocal fluorescence imaging of GSH, Hcy, Cys, and ATP in HepG2 cells. Cells were incubated with different concentrations of APAP (0, 200,500, and 1000 µm), and APAP (1000 µm) + NAC (400 µm) for 12 h. b) Relative pixel intensity of the fluorescence images A1‐E5 in (a). *λ*_ex_ = 405 nm, *λ*_em_ = 420–470 nm for the blue channel, *λ*_ex_ = 458 nm, *λ*_em_ = 500–560 nm for the green channel, and *λ*_ex_ = 488 nm, *λ*_em_ = 540–630 nm for the red channel, and *λ*_ex_ = 514 nm, *λ*_em_ = 580–650 nm for the pink channel. Scale: 25 µm.

As shown in **Figure** **5**, HepG2 cells were divided into three groups. In the control group, cells were treated only with probe **BCR**, resulting in strong fluorescence signals across all channels. In the experimental group, cells were pretreated with varying concentrations of APAP for 12 h to induce graded levels of damage, followed by incubation with probe **BCR**. Fluorescence intensity in all channels diminished progressively with increasing APAP concentrations, reflecting depletion of intracellular GSH, Hcy, Cys, and ATP levels. Additionally, a third group was pre‐incubated with N‐acetylcysteine (NAC), a known antidote for APAP overdose that protects the liver from drug‐induced injury. As expected, NAC intervention restored fluorescence intensity in all channels, suggesting that NAC elevated active sulfur species such as GSH and neutralized toxic APAP metabolites, alleviating oxidative stress and restoring ATP levels.

**Figure 6:**
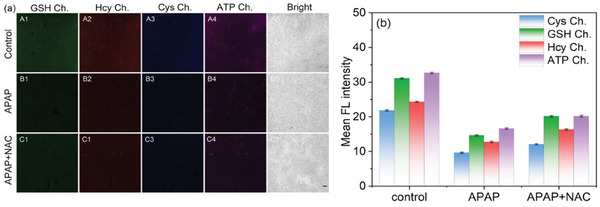
a) Confocal fluorescence imaging of GSH, Hcy, Cys, and ATP in mouse liver tissue with injury. Liver tissues were incubated with probe **BCR** (5 µm) for 45 min. b) The relative pixel intensity of the images in different groups corresponding to (a). Scale: 200 µm.

Building on these findings, we further applied probe **BCR** to investigate APAP‐induced liver injury in a mouse model. Liver tissue sections from APAP‐treated mice exhibited significantly reduced fluorescence in all four channels corresponding to GSH, Hcy, Cys, and ATP, indicating pronounced liver damage (**Figure** **6**). In contrast, tissue sections from NAC‐treated mice showed partial recovery of fluorescence intensity, suggesting mitigation of oxidative stress and restoration of intracellular metabolite levels. In conclusion, probe **BCR** demonstrates excellent potential as a tool for detecting alterations in GSH, Hcy, Cys, and ATP levels during APAP‐induced acute liver injury. Its ability to provide real‐time, multi‐analyte visualization offers a promising avenue for diagnosing and evaluating oxidative stress‐mediated liver injury, including APAP‐induced damage.

## 3 Conclusion

In this study, we successfully designed and synthesized a multifunctional fluorescent probe, **BCR**, by linking a coumarin derivative to a rhodamine derivative through a piperazine bridge. This design strategy yielded a fluorophore with outstanding optical properties, including high selectivity and sensitivity for detecting GSH, Hcy, Cys, and ATP. Experimental validation in cellular models and zebrafish demonstrated that probe **BCR** could specifically and sensitively respond to endogenous GSH, Hcy, Cys, and ATP, enabling real‐time, dynamic monitoring of their intracellular concentrations. Notably, **BCR** was effectively utilized to visualize biomolecular changes in a PTZ‐induced zebrafish epilepsy model, capturing dynamic fluctuations of these biomarkers associated with oxidative stress and energy metabolism. Furthermore, probe **BCR** was successfully applied in an APAP‐induced mouse liver injury model, revealing the metabolic dynamics of GSH, Hcy, Cys, and ATP during drug‐induced liver injury. These findings establish probe **BCR** as a versatile and reliable tool for multi‐target detection in complex biological systems. Its ability to provide dynamic, real‐time imaging of key biochemical markers offers valuable insights into the molecular mechanisms underlying various diseases. This innovative probe holds great potential for advancing the study of disease pathogenesis, oxidative stress, and therapeutic interventions.

## 4 Experimental Section

### General Information and Methods

The chemicals were purchased from Aladdin or Bide Pharmatech Ltd. (Shanghai, China) and used without further purification. Solvents used were purified by standard methods prior to use. Ultrapure water (18.25 MΩ·cm) was used throughout all experiments (Aquapro water ultrapurification system). All reactions were magnetically stirred and monitored by thin‐layer chromatography (TLC). Column chromatography was conducted over silica gel (200–300 mesh). UV–vis absorption spectra were collected on a UV‐2450 and UV‐2600i spectrophotometer (Shimadzu Co., Japan). Fluorescence spectra were recorded on a Hitachi F‐7100 spectrophotometer (Hitachi Ltd, Japan) with a 1 cm standard quartz cell (PMT voltage, 700 V; Scan speed, 1200 nm min^−1^; Delay, 0 s; Response, 2 s). The visual pictures were captured by a smartphone's built‐in camera. ^1^H NMR and ^13^C NMR spectra were obtained with tetramethyl silane (TMS) as the internal standard on a BRUKER AVANCE‐500 spectrometer and chemical shifts (*δ*) were expressed in ppm and coupling constants (*J*) in Hertz. IR spectrum was recorded on NEXUS as KBr pellets and the data was reported in cm^−1^. High‐resolution mass spectra were performed using the Agilent 1290/6545 UHPLC‐QTOF/MS mass spectrometer system. All of the fresh hippocampal tissue slices (thickness: 20 µm) were obtained on CM1860UV (Leica, Germany). All of the cells and zebrafish imaging experiments were conducted on a fluorescence confocal microscope system (Leica SP8, Germany).

### Synthesis of the Probe BCR

To a solution of the compound (E)‐4‐((3‐(2‐(benzo[d]thiazol‐2‐yl)‐2‐cyanovinyl)‐4‐(butylthio)‐2‐oxo‐2H‐chromen‐7‐yl)(ethyl)amino)butanoic acid (96.0 mg, 175.28 µmol) in 8 mL anhydrous dichloromethane was added 4‐dimethylaminopyridine (DMAP) (2.1 mg, 17.53 µmol), and the mixture was stirred at room temperature for 10 min. Subsequently, 2‐(2‐((2‐aminoethyl)amino)ethyl)‐3',6'‐bis(diethylamino)spiro[isoindoline‐1,9'‐xanthen]‐3‐one (92.5 mg, 175.28 µmol) and 1‐(3‐dimethylaminopropyl)‐3‐ethylcarbodiimide hydrochloride (EDCI) (50.4 mg, 262.92 µmol) were added, and the reaction mixture was stirred for 2 h. The reaction progress was monitored by TLC. Upon completion, the crude product was purified by column chromatography to afford the red product, probe BCR (110.0 mg, 59.35%). ^1^H NMR (500 MHz, CDCl_3_) δ 8.39 (s, 1H), 8.07 (d, *J* = 8.1 Hz, 1H), 792–7.87 (m, 2H), 7.81–7.79 (m, 1H), 7.69–7.64 (m, 1H), 7.53–7.36 (m, 5H), 7.09–7.07 (m, 1H), 6.75–6.72 (m, 1H), 6.51 (d, *J* = 2.5 Hz, 1H), 6.42 (d, *J* = 8.9 Hz, 2H), 6.36 (d, *J* = 2.5 Hz, 2H), 6.28–6.25 (m, 2H), 3.51–3.41 (m, 6H), 3.37 (d, *J* = 5.1 Hz, 2H), 3.31 (d, *J* = 7.1 Hz, 8H), 2.95–2.90 (m, 4H), 2.68–2.65 (m, 2H), 2.42–2.39 (m, 2H), 2.02–1.96 (m, 2H), 1.59–1.52 (m, 2H), 1.38–1.32 (m, 2H), 1.21 (d, *J* = 7.0 Hz, 3H), 1.16–1.13 (m, 12H), 0.83 (d, *J* = 7.3 Hz, 3H). IR (KBr pellet, cm^−1^): 3436, 2968, 2920, 1611, 1577, 1546, 1513.08, 1468, 1409, 1381, 1302, 1263, 1221, 1148, 1117, 1075, 1050, 879, 823, 786, 761, 700, 649, 486, 428. ^13^C NMR (700 MHz, CDCl_3_) δ 173.23, 170.21, 163.15, 157.68, 157.29, 155.53, 153.93, 153.69, 153.43, 152.80, 149.18, 141.61, 135.28, 133.38, 130.04, 129.47, 128.45, 126.84, 126.59, 125.89, 124.15, 123.78, 122.94, 121.71, 116.12, 115.77, 110.53, 110.36, 110.16, 108.48, 103.95, 97.87, 97.32, 66.45, 53.55, 50.30, 48.29, 47.74, 45.64, 44.46, 38.63, 37.30, 37.05, 32.80, 32.07, 29.78, 22.98, 21.76, 13.57, 12.67, and 12.38. High‐resolution mass spectrum (HRMS, ESI^+^), calculated for C_61_H_69_N_8_O_5_S_2_^+^ [M+H]^+^ calcd: 1057.4827, found: 1057.4790 (Scheme S1, Supporting Information).

### Cell Culture

HeLa, SH‐SY5Y, and HepG2 cells were obtained from the China Center for Type Culture Collection (Wuhan, China). The cells were cultured in Dulbecco's Modified Eagle's Medium (DMEM) supplemented with 10% (v/v) heat‐inactivated fetal bovine serum (FBS) (Gibco BRL, Grand Island, NY, USA) and streptomycin‐penicillin (100 U mL^−1^ and 100 µg mL^−1^, respectively). The cultures were maintained in a humidified incubator at 37 °C with 5% CO₂ and 95% air.

### Imaging of Endogenous and Exogenous Cys, Hcy, GSH, and ATP in Living Cells

For monitoring endogenous Cys, Hcy, GSH, and ATP in living cells, HeLa, SH‐SY5Y, and HepG2 cells were incubated with probe BCR (5 µm in serum‐free DMEM buffer containing 0.5% DMSO (v/v)) for 60 min. After incubation, the cells were washed three times with PBS and imaged. To detect exogenous Cys, Hcy, GSH, and ATP, the cells were pretreated with N‐ethylmaleimide (NEM) (0.1 mm, a biothiol scavenger) and Oligomycin A (Omy A) (50 µm, an ATP scavenger) respectively. Subsequently, the cells were incubated with Cys (0.2 mm), Hcy (0.2 mm), GSH (0.2 mm), NaHSO₃ (250 µm), NaHS (250 µm), and Na₂Sn (n ≥ 2) (250 µm), respectively, followed by treatment with probe BCR (5 µm). After incubation, the cells were washed three times with PBS and imaged using a fluorescence confocal microscope (Leica SP8, Germany). All experiments were repeated three times, with imaging parameters as follows: *λ*_ex_ = 405 nm, *λ*_em_ = 420–470 nm for the blue channel, *λ*_ex_ = 458 nm, *λ*_em_ = 500–560 nm for the green channel, and *λ*_ex_ = 488 nm, *λ*_em_ = 540–630 nm for the red channel, and *λ*_ex_ = 514 nm, *λ*_em_ = 580–650 nm for the pink channel).

### Imaging of GSH, Hcy, Cys, and ATP in Pentylenetetrazole (PTZ)‐Induced Cell Injury

SH‐SY5Y cells were cultured in a confocal dish. Experimental Group: When the cell adhesion rate reached 70–80%, PTZ was added to the petri dish at final concentrations of 0.1, 0.3, and 0.5 mm. The cells were incubated for 12 h, washed three times with PBS solution, and then treated with probe **BCR** (5 µM) for 45 min. After incubation, the cells were washed three times with PBS solution before fluorescence imaging. Control Group: Probe **BCR** (5 µm) was directly added to the culture dish, incubated with the cells for 45 min, and then washed three times with PBS solution before fluorescence imaging. All experiments were performed in triplicate.

### Imaging of Endogenous Cys, Hcy, GSH, and ATP in Zebrafish

Prof. Yun Deng (Zebrafish Genetics Laboratory, College of Life Science, Hunan Normal University, Changsha, China) provided the normal zebrafish and diabetes model zebrafish (The diabetes models of zebrafish were constructed according to ref. ^[^ ^48^ ^]^). All animal procedures were performed in accordance with the guidelines and approved by the Animal Ethics Committee of Hunan Normal University (No. 2022–161). For the detection of endogenous Cys, Hcy, GSH, and ATP in vivo, 2/4‐day‐old zebrafish was prepared. The zebrafish was incubated with probe **BCR** (5 µm) in E3 embryo media (containing 0.5% DMSO, v/v) at 28 °C for 60 min, then imaged. All the zebrafish were terminally anesthetized using MS222, and fluorescence imaging was conducted on a fluorescence confocal microscope system (Leica SP8, Germany). All experiments were repeated three times. (*λ*_ex_ = 405 nm, *λ*_em_ = 420–470 nm for the blue channel, *λ*_ex_ = 458 nm, *λ*_em_ = 500–560 nm for the green channel, and *λ*_ex_ = 488 nm, *λ*_em_ = 540–630 nm for the red channel, and *λ*_ex_ = 514 nm, *λ*_em_ = 580–650 nm for the purple channel).

### Imaging of GSH, Hcy, Cys, and ATP in Zebrafish Epileptic Model Induced by PTZ

Zebrafish were cultured in six‐well plates. Experimental group, 6mm PTZ was added and incubated for 3, 6, and 12 h respectively, and then washed with PBS buffer solution three times, and then probe **BCR** (5 µm) was added and incubated with zebrafish for 45 min. After washing with PBS buffer solution three times, Zebrafish was placed on the slide surface for imaging with tissue fixation solution. Control group: Directly added probe **BCR** (5 µm) and zebrafish were incubated for 45 min, washed three times with PBS buffer solution, and then placed on the slide surface with tissue fixation solution for imaging.

## Conflict of Interest

The authors declare no conflict of interest.

## Supporting information



Supporting Information

## Data Availability

The data that support the findings of this study are available in the supplementary material of this article.
