# Supplementary material for: Multifunctional Fluorescent Probe for Simultaneous Detection of ATP, Cys, Hcy, and GSH: Advancing Insights into Epilepsy and Liver Injury
Source: Adv Sci (Weinh). 2025 Jan 30;12(11):2415882. doi: 10.1002/advs.202415882 (PMC11923924; doi:10.1002/advs.202415882)
Supplement: Supplementary file 1 — Supporting Information [file ADVS-12-2415882-s001.docx]

Supporting Information

Multifunctional Fluorescent Probe for Simultaneous Detection of ATP, Cys, Hcy, and GSH: Advancing Insights into Epilepsy and Liver Injury

Ting Yu,^a,c#^ Yang Li,^a,b#^ Jing Li,^a,b#^ Yabing Gan,^a,b^ Zhengze Long,^a,b^ Yun Deng,^a,c^* Youyu Zhang,^a,b^ Haitao Li,^a,b^ Peng Yin,^a,b^* and Shouzhuo Yao^b^

^a^ Institute of Interdisciplinary Studies, Hunan Normal University, Changsha, 410081, China.

^b^ Key Laboratory of Chemical Biology and Traditional Chinese Medicine Research (Ministry of Education), College of Chemistry and Chemical Engineering, Hunan Normal University, Changsha, 410081, China.

^c^ College of Life Sciences, Hunan Normal University, Changsha, 410081, China.

Table of Contents

I. Experimental Section ………………………………………………………………………………S2

II. Supplementary Spectra and Figures…………………………….………….......………………….S21

III. ^1^H NMR and ^13^C NMR Spectra…………..............................................................................……S46

IV. References…….............................................……..............................……………………………S68

I. Experimental Section

General procedure for fluorescence and UV-visible measurements.

The stock solution of probe (10 mM) was prepared in DMSO. Stock solutions (1×10^-2^ M) of GSH, Hcy Cys, ATP（10 mM）, ^•^OH, NO, ^1^O_2_, O_2_^•-^, H_2_O_2_, NaClO, NaHSO_3_, NaHS, NAC, Ca^2+^, Mg^2+^, Co^2+^, Zn^2+^, Sn^4+^, Ba^2+^, Cd^2+^, Cu^2+^, Cu^+^, Fe^2+^, Fe^3+^, Al^3+^, SO_4_^2-^, CO_3_^2-^, Ac^-^, NO_3_^-^, Asp, Pro, Glu, Tyr, Ser, Val, Ala, Leu, Thr, Dop were prepared in ultrapure water (18.25 MΩ·cm). These stock solutions were further diluted to the required concentration for measurement. Test solutions were prepared as follows: 20 μL of probe solution (1.0 mM) was added into a test tube, and the solution was diluted with PBS buffer containing 40% or 50% DMSO (v/v), then proper analyte’s solution was added into the test tube. Absorption and fluorescence spectra were recorded in an indicated time at 25 °C.

MTT assay for the cell cytotoxicity.

Cell cytotoxicity was evaluated by MTT (3-(4,5-dimethyl-2-thiazolyl)-2,5-diphenyltetrazolium bromide) assay. Cells were cultivated in a 96-well plate until 60–70% confluence, and incubated with different concentrations of probe (0–10 μM) for 24 h. Then 20 μL MTT, (5 mg/mL) was added and incubated for 4 h at 37 °C. After MTT was washed, 150 μL DMSO was added. Absorbance was measured at 570 nm with a multi-function microplate reader (SpectraMax i3, USA). All of experiments were repeated six times, and the data were presented as the percentage of control cells.

Calculation of the limit of detection (LOD)

LOD = 3σ*/S*

σ: the standard deviation of the blank solution.

𝑥̅ is the mean of the blank measures; 𝑥_𝑖_ is the values of blank measures; n is the number of tested blank measure (n = 10)

*S*: the slope of the linear calibration plot between the fluorescence emission intensity and the concentration of GSH, Hcy, Cys, and ATP.

Scheme S1. Synthesis of compounds 2-7 and Probe BCR.

Synthesis of compounds 2.

The 3.01 g (21.93 mmol) 3-(ethylamino)phenol (synthesized according to reference 1) was dissolved in 50 mL anhydrous acetonitrile and 5.87 g (26.31 mmol) tert-butyl 4-bromobutyrate was added. Then 3.04 g (23.02 mmol) of ammonium hydrogen phosphate and 1.82 g (10.96 mmol) of potassium iodide were added and the reaction mixture was stirred overnight at 95 ºC. After the reaction was monitored by TLC, the solvent acetonitrile was removed under vacuum reduction, and the crude product was purified by silica gel chromatography to obtain compound 2 (3.34 g, 54.52%). ^1^H NMR (500 MHz, CDCl_3_) δ 7.06-7.03 (m, 1H), 6.27-6.25 (m, 1H), 6.25-6.20 (m, 1H), 6.17-6.14 (m, 1H), 3.34 3.30 (m, 2H), 3.26 3.23 (m, 2H), 2.29 2.26 (m, 2H), 1.90 1.84 (m, 2H), 1.47 (s, 9H), 1.25-1.22 (m, 3H). ^13^C NMR (126 MHz, CDCl_3_) δ173.18, 157.04, 149.41, 130.13, 104.58, 102.74, 98.91, 80.75, 49.56, 45.01, 32.91, 28.14, 22.89, 12.25.

Synthesis of compounds 3.

3.34 g (11.96 mmol) compound 2 was dissolved in 30 mL of newly prepared toluene solution, then 5.53 g (11.96 mmol) of bis (2, 4, 6-trichlorophenyl) malonate was added, and the reaction mixture was stirred by reflux at 110 ºC. After the reaction was monitored by TLC, the solution was cooled to room temperature and then filtered to obtain solid compound 3 (2.34 g, 56.34%). ^1^H NMR (500 MHz, CDCl_3_) δ 10.97 (s, 1H), 7.84 (d, J = 9.2 Hz, 1H), 6.77 - 6.75 (m, 1H), 6.53 (d, J = 2.5 Hz, 1H), 5.49 (s, 1H), 3.50 - 3.40 (m, 4H), 2.32 - 2.29 (m, 2H), 1.92 - 1.89 (m, 2H), 1.47 (s, 9H), 1.25 - 1.22 (m, 3H). ^13^C NMR (126 MHz, CDCl_3_) δ 171.98, 167.77, 163.73, 163.49, 161.19, 155.65, 153.46, 125.70, 110.70, 100.97, 97.32, 91.83, 88.39, 80.99, 49.87, 45.59, 32.23, 28.14, 22.43, 12.12.

Synthesis of compounds 4.

Under argon, fresh distilled DMF (0.5 mL) was slowly added dropwise to POCl_3_ (2.0 mL) at room temperature and stirred for 30 min to yield an orange-red solution. Then a portion of (compound 3, 2.34 g,6.74 mmol, dissolved in 10 mL fresh distilled DMF) was added dropwise to the above solution and a scarlet suspension was yielded. The mixture was stirred at 80 ºC for overnight until the reaction was completed. Then reaction mixture was slowly poured into 200 mL of ice water. NaOH solution (20%) was added to adjust the pH to 6–7. The mixture was extracted with ethyl acetate, the combined ethyl acetate extract was washed and dried, then vacuum evaporated, and the residue was purified by silica gel chromatography to obtain compounds 4.(1.56 g，68.57%）。^1^H NMR (500 MHz, DMSO-*d_6_*) δ 10.04 (d, *J* = 2.9 Hz, 1H), 7.76 - 7.73 (m, 1H), 6.91 (d, *J* = 9.2 Hz, 1H), 6.65 (s, 1H), 3.52 - 3.49 (m, 2H), 3.45 - 3.41 (m, 2H), 2.35 - 2.32 (m, 2H), 1.79 - 1.76 (m, 2H), 1.15 - 1.12 (m, 3H). ^13^C NMR (126 MHz, DMSO-d_6_) δ 186.74, 174.64, 159.68, 156.40, 154.25, 152.62, 129.23, 111.65, 110.62, 107.22, 96.78, 49.75, 45.50, 30.88, 22.57, 12.67.

Synthesis of compounds 5

Dissolve 1.56g (4.62 mmol) compound 4 in 20 mL anhydrous dichloromethane solution, stir the reaction mixture at room temperature for 2 minutes, then add 701.1 mg (6.93 mmol) triethylamine and 499.8 mg (5.54 mmol) N-butyl mercaptan, and react at room temperature for 8 hours away from light. After the reaction was completed by TLC monitoring, the mixture was concentrated into a crude product, which was further purified by column chromatography to obtain compound 5 (1.01g, 55.86%).^1^H NMR (500 MHz, CDCl_3_) δ 10.26 (s, 1H),7.99 (d, *J* = 9.4 Hz, 1H), 6.66 - 6.64 (m, 1H), 6.42 (d, *J* = 2.6 Hz, 1H), 3.45 - 3.40 (m, 4H), 3.00 - 2.97 (m, 2H), 2.44 - 2.42 (m, 2H), 1.94 - 1.91 (m, 2H), 1.56 - 1.50 (m, 2H), 1.35 - 1.30 (m, 2H), 1.20 - 1.17 (m, 3H), 0.84 - 0.80 (m, 3H). ^13^C NMR (126 MHz, CDCl_3_) δ188.40, 177.28, 163.08, 160.48, 155.75, 153.40, 129.80, 113.62, 110.35, 110.11, 97.09, 49.83, 45.69, 38.42, 31.99, 30.76, 22.21, 21.78, 13.56, 12.34.

Synthesis of compounds 6

The 100.0 mg (255.44 μmol) compound 5 was dissolved in 8 mL anhydrous ethanol, then 44.5 mg (255.44 μmol) benzothiazole acetonitrile was added, and the reaction mixture was stirred at 30 ºC. After the reaction was completed by TLC monitoring, it was observed that solid was formed at the bottom of the reaction bottle. The reaction liquid was cooled and filtered, and the solid was washed with a small amount of anhydrous ethanol to obtain compound 6 (96.0 mg, 68.62%).^1^H NMR (500 MHz, CDCl_3_) δ 8.37 (d, *J* = 3.8 Hz, 1H), 8.07 (d, *J* = 8.1 Hz, 1H), 7.91 - 7.87 (m, 2H), 7.51 - 7.48 (m, 1H), 7.42 (d, *J* = 7.8 Hz, 1H), 6.69 (d, *J* = 7.0 Hz, 1H), 6.52 (s, 1H), 3.47 - 3.42 (m, 4H), 2.97 - 2.91 (m, 2H), 2.45 (d, *J* = 6.5 Hz, 2H), 1.96 - 1.94(m, 2H), 1.57 - 1.53 (m, 2H), 1.34 - 1.31 (m, 2H), 1.23 - 1.20 (m, 3H), 0.82 (d, *J* = 7.8 Hz, 3H).

Synthesis of the probe BCR.

Dissolve 96.0 mg (175.28 μmol) compound 6 in 8 mL anhydrous DCM, add 2.1 mg (17.53 μmol) 4-dimethylaminopyridine (DMAP), stir and react at room temperature for 10 min. Then 92.5 mg (175.28 μmol) of compound 7 (synthesis in reference) and 50.4 mg (262.92 μmol) of 1- (3-dimethylaminopropyl) -3-ethylcarbodiimide hydrochloride (EDCI) were added and continued to stir for 2 hours. After the reaction was completely monitored by TLC, the crude product was purified by column chromatography to obtain the red product (Probe BCR, 110.0 mg, 59.35%). ^1^H NMR (500 MHz, CDCl_3_) δ 8.39 (s, 1H), 8.07 (d, *J* = 8.1 Hz, 1H), 792 - 7.87 (m, 2H), 7.81 - 7.79 (m, 1H), 7.69 - 7.64 (m, 1H), 7.53 - 7.36 (m, 5H), 7.09 - 7.07 (m, 1H), 6.75 - 6.72 (m, 1H), 6.51 (d, *J* = 2.5 Hz, 1H), 6.42 (d, *J* = 8.9 Hz, 2H), 6.36 (d, *J* = 2.5 Hz, 2H), 6.28 - 6.25 (m, 2H), 3.51 - 3.41 (m, 6H), 3.37 (d, *J* = 5.1 Hz, 2H), 3.31 (d, *J* = 7.1 Hz, 8H), 2.95 - 2.90 (m, 4H), 2.68 - 2.65 (m, 2H), 2.42 - 2.39 (m, 2H), 2.02 - 1.96 (m, 2H), 1.59 - 1.52 (m, 2H), 1.38 - 1.32 (m, 2H), 1.21 (d, *J* = 7.0 Hz, 3H), 1.16 - 1.13 (m, 12H), 0.83 (d, *J* = 7.3 Hz, 3H). IR (KBr pellet, cm^–1^): 3436, 2968, 2920, 1611, 1577, 1546, 1513.08, 1468, 1409, 1381, 1302, 1263, 1221, 1148, 1117, 1075, 1050, 879, 823, 786, 761, 700, 649, 486, 428. ^13^C NMR (700 MHz, CDCl_3_) δ 173.23, 170.21, 163.15, 157.68, 157.29, 155.53, 153.93, 153.69, 153.43, 152.80, 149.18, 141.61, 135.28, 133.38, 130.04, 129.47, 128.45, 126.84, 126.59, 125.89, 124.15, 123.78, 122.94, 121.71, 116.12, 115.77, 110.53, 110.36, 110.16, 108.48, 103.95, 97.87, 97.32, 66.45, 53.55, 50.30, 48.29, 47.74, 45.64, 44.46, 38.63, 37.30, 37.05, 32.80, 32.07, 29.78, 22.98, 21.76, 13.57, 12.67, 12.38.High resolution mass spectrum (HRMS, ESI^+^), calculated for C_61_H_69_N_8_O_5_S_2_^+^ [M+H]^+^ calcd: 1057.4827, found: 1057.4790.

Table S1. Comparison of probe BCR with reported fluorescent probes

| Probe | *λ*_ex_/*λ*_em_ | Selectivity | Application | Ref. |
| --- | --- | --- | --- | --- |
|  | ATP: λ_ex_/λ_em_ = 401/590 nm  H_2_O_2_: λ_ex_/λ_em_ = 380/470nm | ATP, H_2_O_2_ | *In vitro, cell* | *J. Am. Chem. Soc.* 2020, 142, 7532−7541 |
|  | ATP: λ_ex_/λ_em_ = 520/587 nm  ONOO^−^: λ_ex_/λ_em_ = 450/562 nm | ATP, ONOO^−^ | *cells* | *J. Am. Chem. Soc.* 2022, 144, 174−183 |
|  | ATP: λ_ex_/λ_em_ = 520/588 nm  O_2_^•−^: λ_ex_/λ_em_ = 380/470 nm | ATP, O_2_^•−^ | *Cells, Mouse livers* | *J. Am. Chem. Soc.* 2023, 145, 19662−19675 |
|  | ATP: λ_ex_/λ_em_ = 530/560 nm  H_2_S: λ_ex_/λ_em_ =  440/530 nm | ATP, H_2_S | *cells, zebrafish* | *Chem. Eng. J.* 442 (2022) 136141 |
|  | ATP: λ_ex_/λ_em_ = 445/577 nm  H_2_S: λ_ex_/λ_em_ =  450/528 nm | ATP, H_2_S | *Cells, Mouse* | *Anal. Chem.* 2022, 94, 11573−11581 |
|  | ATP: λ_ex_/λ_em_ = 520/588 nm  H_2_S: λ_ex_/λ_em_ =  440/534 nm | ATP, Fe^2+^ | *Cell, zebrafish* | *Sens. Actuators B Chem.* 2023, 393, 134288 |
|  | ATP: λ_ex_/λ_em_ = 413/633 nm  GSH: λ_ex_/λ_em_ = 330/467 nm | ATP, GSH | *cells, zebrafish* | *Sens. Actuators B Chem.* 2024, 412, 135772 |
|  | ATP: λ_ex_/λ_em_ = 520/587  GSH: λ_ex_/λ_em_ = 455/529 nm  Hcy: λ_ex_/λ_em_ = 493/555 nm  Cys: λ_ex_/λ_em_ = 375 nm/455 nm | ATP, GSH, Hcy, and Cys | *In vitro*, cells, zebrafish, Liver tissue | *This work* |

II. Supplementary Spectra and Figures


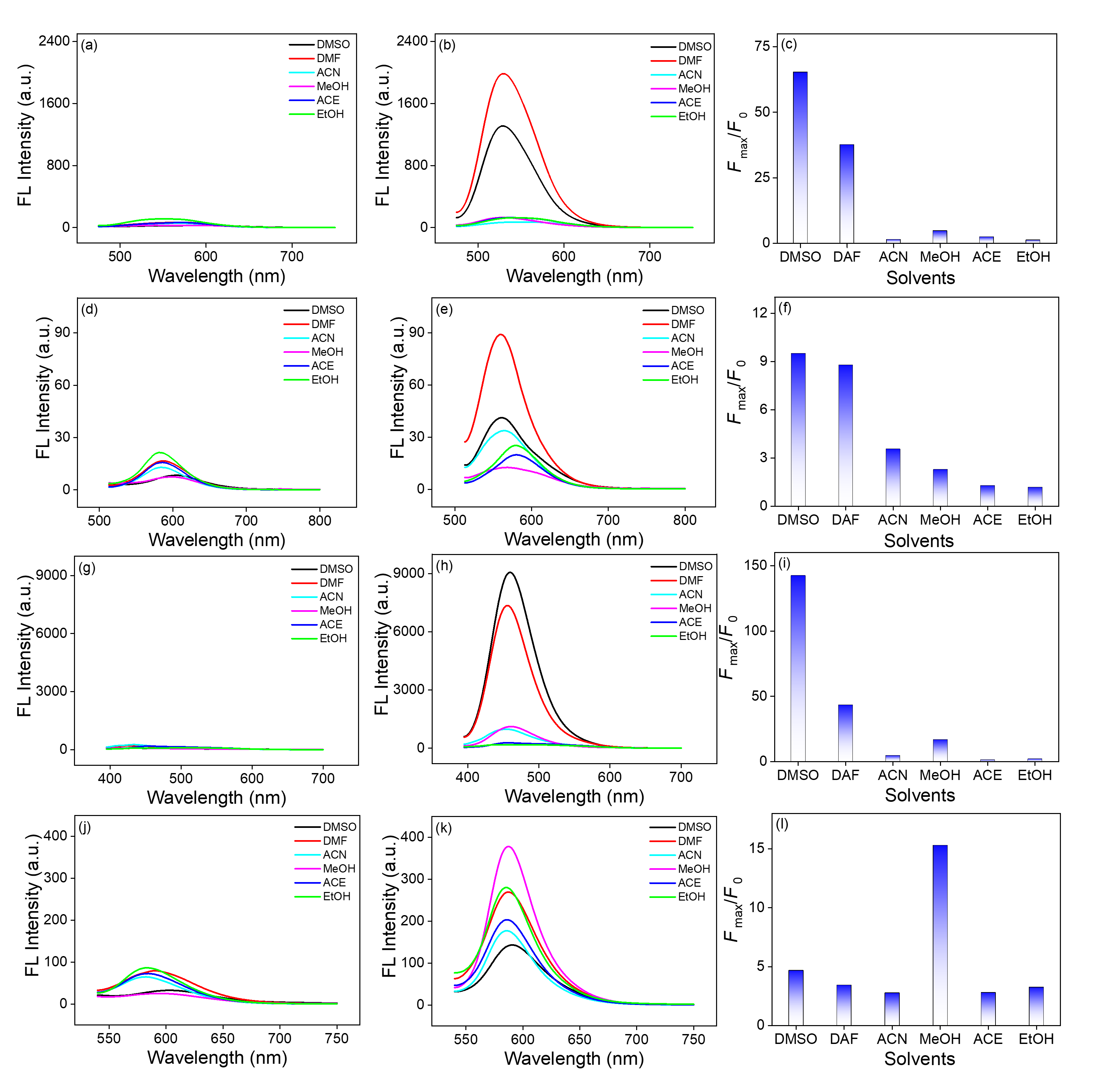


Figure S1. Fluorescence spectra of BCR (10 μM) in the absence (a), (d), (g), (j) and presence (b), (e), (h), (k) of GSH/Hcy/Cys (100 μM) and ATP (10 mM) in different organic phase solvents/PBS (10 mM, pH = 7.4, v/v, 5/5) at 25 °C for 30 min. The ratio of fluorescence intensity change corresponding to probe BCR (10 µM) in the presence (c), (f), (i), (l) of the same organic phase solvent /PBS. Organic phase solvent: 1. DMSO (dimethyl sulfoxide); 2. DMF (N, N-dimethylformamide); 3. ACN (acetonitrile); 4. MeOH (methanol); 5. ACE (Acetone); 6. EtOH (ethanol). Slit (nm): 2.5/5 (GSH/Hcy/ ATP), 5/5 (Cys). (a) *λ*_ex_ = 455 nm, (d) *λ*_ex_ = 493 nm, (g) *λ*_ex_ = 375 nm, (j) *λ*_ex_ = 520 nm.


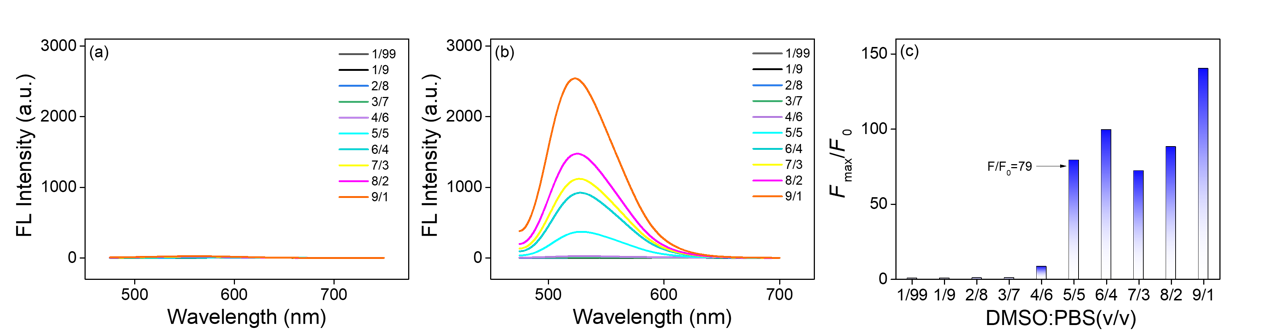


Figure S2. Fluorescence spectra of probe BCR (10 μM) in the absence (a) and presence (b) of GSH (100 μM) in various volume ratio of DMSO/PBS (10 mM, pH = 7.4, v/v) at 25 °C for 30 min. (c) The corresponding fluorescence intensity changes. λ_ex_ = 455 nm, slits (nm): 2.5/5.


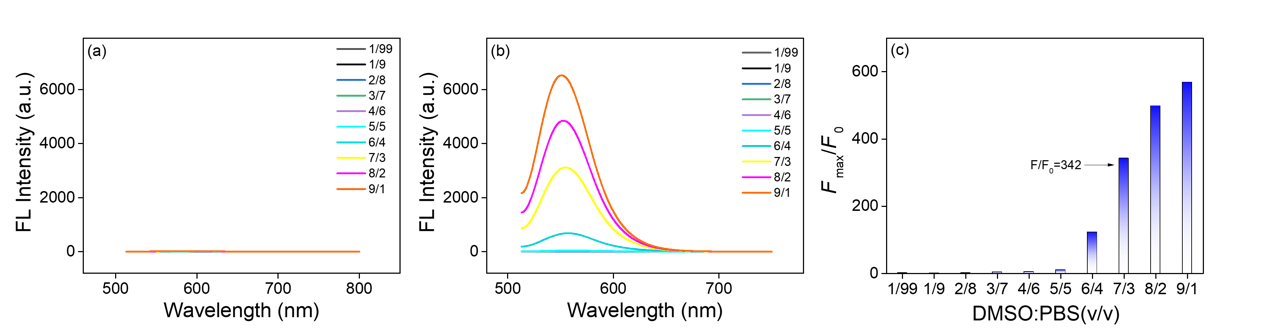


Figure S3. Fluorescence spectra of probe BCR (10 μM) in the absence (a) and presence (b) of Hcy (100 μM) in various volume ratio of DMSO/PBS (10 mM, pH = 7.4, v/v) at 25 °C for 30 min. (c) The corresponding fluorescence intensity changes. λ_ex_ = 493 nm, slits (nm): 2.5/5.


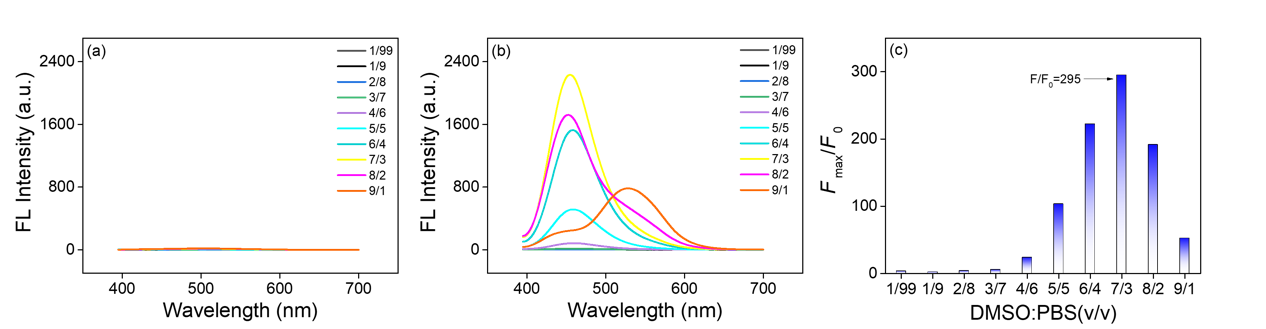


Figure S4. Fluorescence spectra of probe BCR (10 μM) in the absence (a) and presence (b) of Cys (100 μM) in various volume ratio of DMSO/PBS (10 mM, pH = 7.4, v/v) at 25 °C for 30 min. (c) The corresponding fluorescence intensity changes. λ_ex_ = 375 nm, slits (nm): 2.5/2.5.


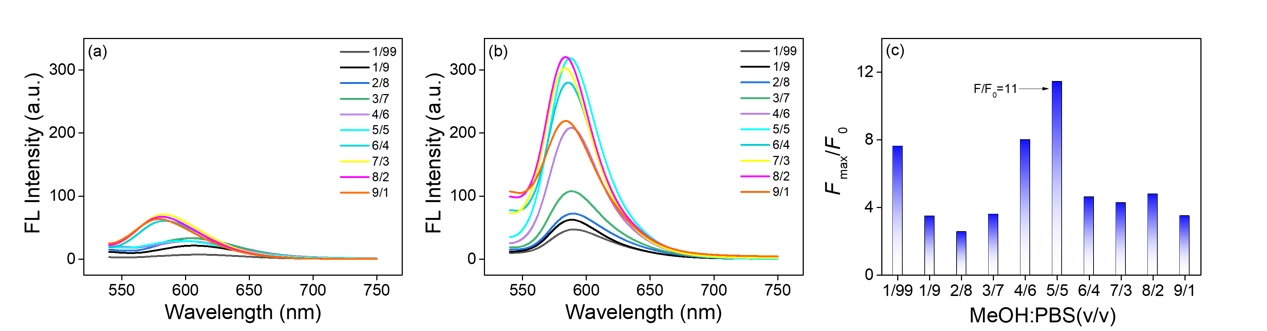


Figure S5. Fluorescence spectra of probe BCR (10 μM) in the absence (a) and presence (b) of ATP (10 mM) in various volume ratio of MeOH/ PBS (10 mM, pH = 7.4, v/v) at 25 °C for 30 min. (c) The corresponding fluorescence intensity changes. λ_ex_ = 520 nm, slits (nm): 5/5.


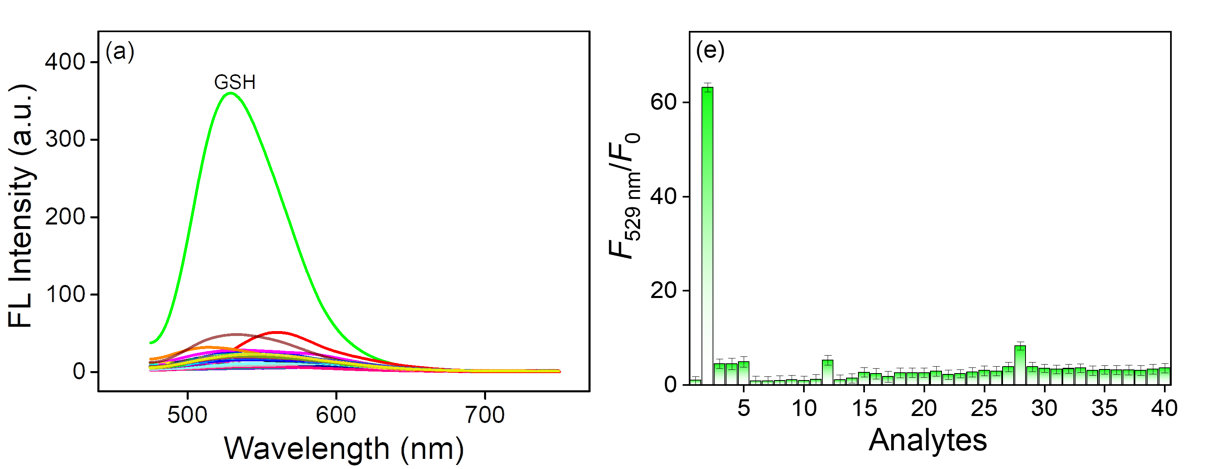


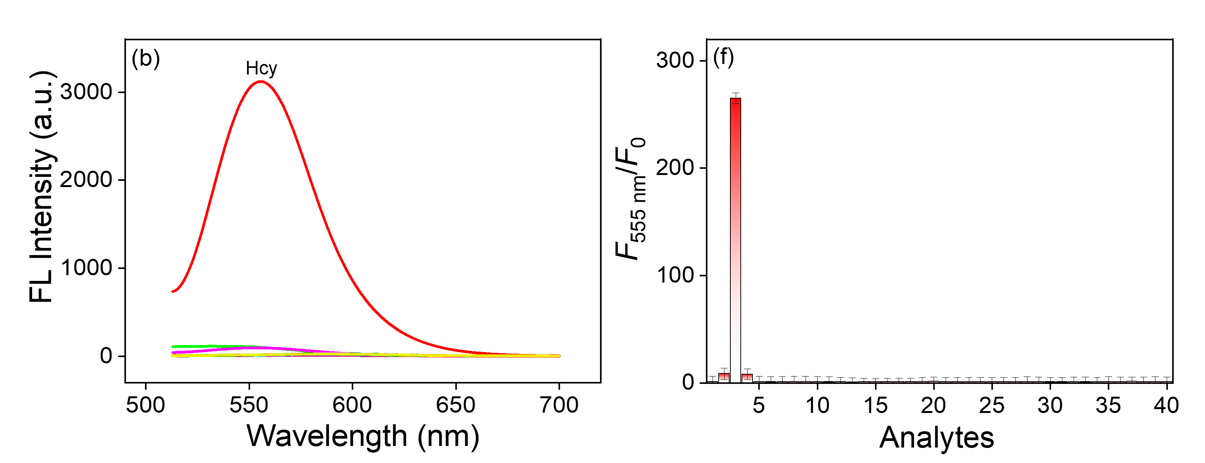


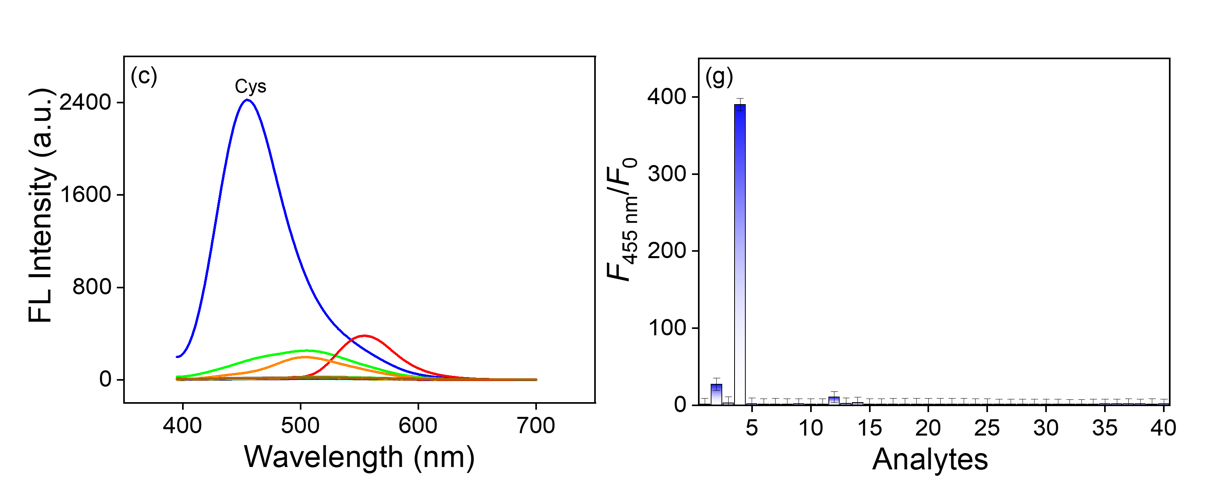


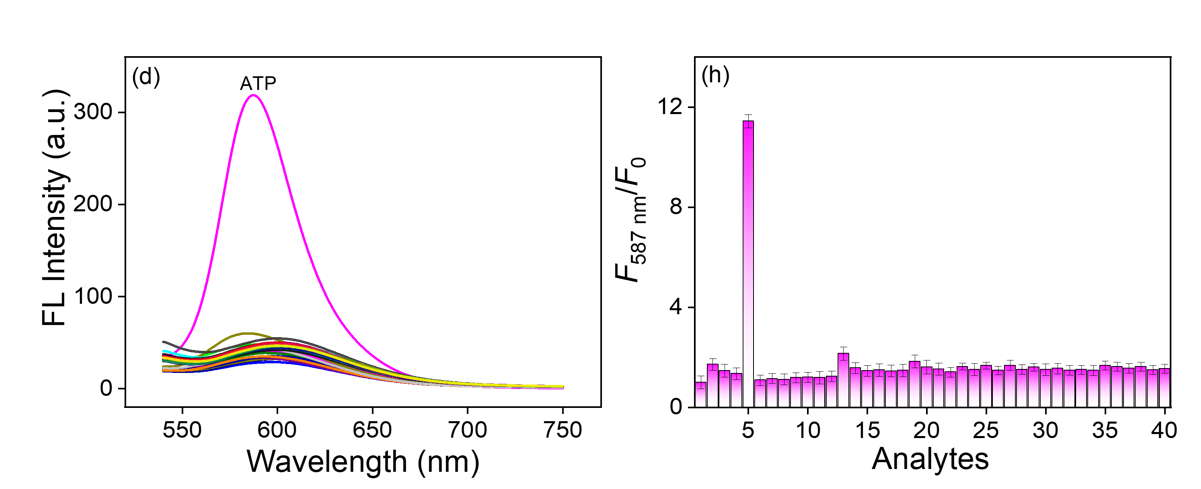


Figure S6. (a–d) Fluorescence spectra of probe BCR (10.0 μM) after the addition of different analytes (100 μM). (e–h) Histogram representing the fluorescence intensity changes of probe BCR (10 µM) at (e) 529 nm, (f) 555 nm, (g) 455 nm, and (h) 587 nm in the presence of different analytes, respectively. Each spectrum was recorded after 20 min. Analytes: 1. BCR, 2. GSH, 3. Hcy , 4. Cys, 5. ATP, 6. •OH, 7. NO, 8. ^1^O_2_, 9. O_2_^•-^, 10. H_2_O_2_, 11. NaClO, 12. NaHSO_3_, 13. NaHS, 14. NAC, 15. Ca^2+^, 16. Mg^2+^, 17. Co^2+^, 18. Zn^2+^, 19. Sn^4+^, 20. Ba^2+^, 21. Cd^2+^, 22. Cu^2+^, 23. Cu^+^, 24. Fe^2+^, 25. Fe^3+^, 26. Al^3+^, 27. SO_4_^2-^, 28. CO_3_^2-^, 29. Ac^-^, 30. NO_3_^-^, 31. Asp, 32. Pro, 33. Glu, 34. Tyr, 35. Ser, 36. Val, 37. Ala, 38. Leu, 39. Thr, 40. Dop. (a) *λ*_ex_ = 455 nm, (b) *λ*_ex_ = 493 nm, (c) *λ*_ex_ = 375 nm, (d) *λ*_ex_ = 520 nm.


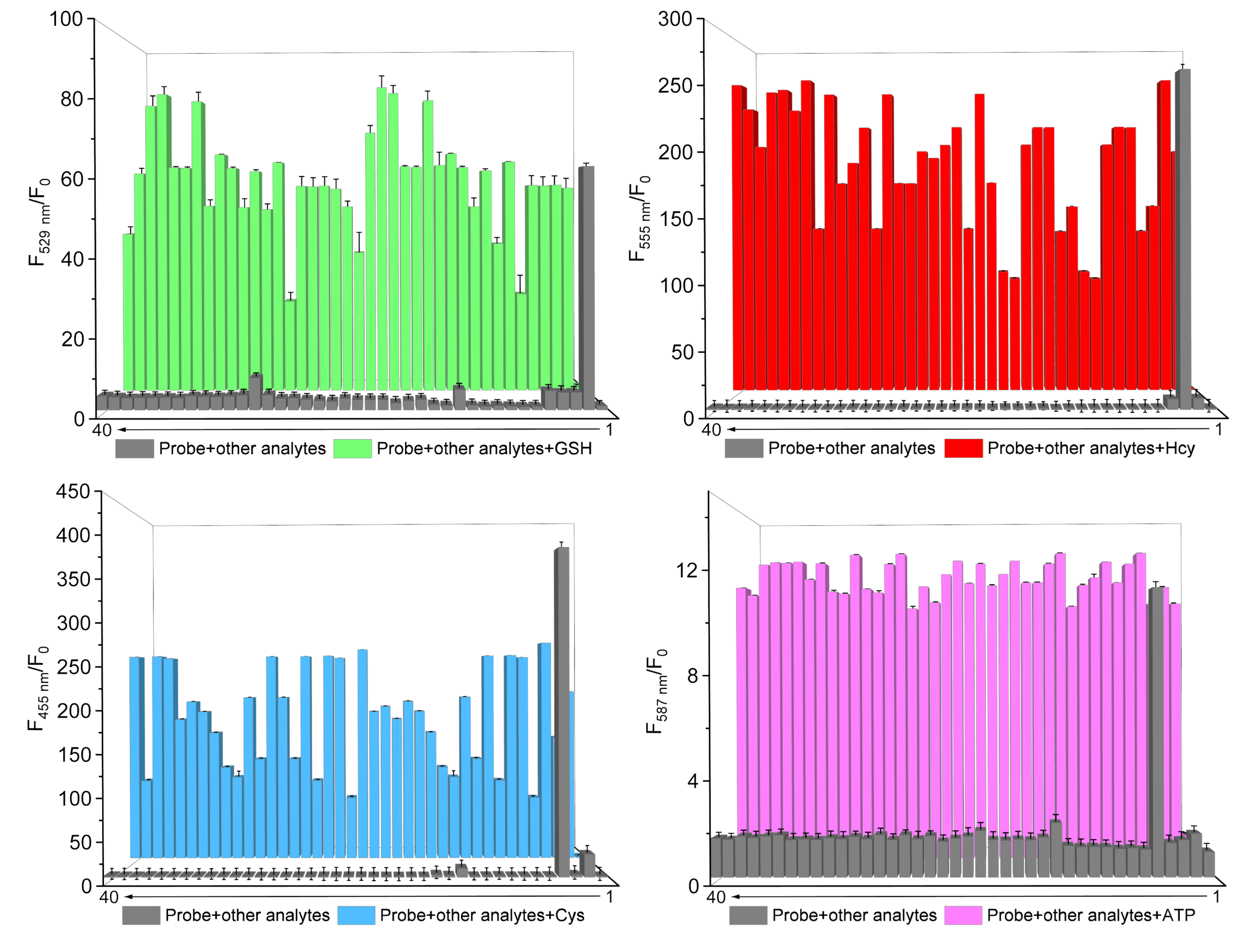


Figure S7. Fluorescence response of BCR (10 μM) with addition of (a) GSH, (b) Hcy,(c) Cys,(d) ATP,(350 μM) in presence of different analytes (100 μM). Each spectrum was recorded after 20 min. Analytes: 1. probe, 2. GSH, 3. Hcy , 4. Cys, 5. ATP, 6. •OH, 7. NO, 8. ^1^O_2_, 9. O_2_^•-^, 10. H_2_O_2_, 11. NaClO, 12. NaHSO_3_, 13. NaHS, 14. NAC, 15. Ca^2+^, 16. Mg^2+^, 17. Co^2+^, 18. Zn^2+^, 19. Sn^4+^, 20. Ba^2+^, 21. Cd^2+^, 22. Cu^2+^, 23. Cu^+^, 24. Fe^2+^, 25. Fe^3+^, 26. Al^3+^, 27. SO_4_^2-^, 28. CO_3_^2-^, 29. Ac^-^, 30. NO_3_^-^, 31. Asp, 32. Pro, 33. Glu, 34. Tyr, 35. Ser, 36. Val, 37. Ala, 38. Leu, 39. Thr, 40. Dop. (a) *λ*_ex_ = 455 nm, (b) *λ*_ex_ = 493 nm, (c) *λ*_ex_ = 375 nm, (d) *λ*_ex_ = 520 nm.


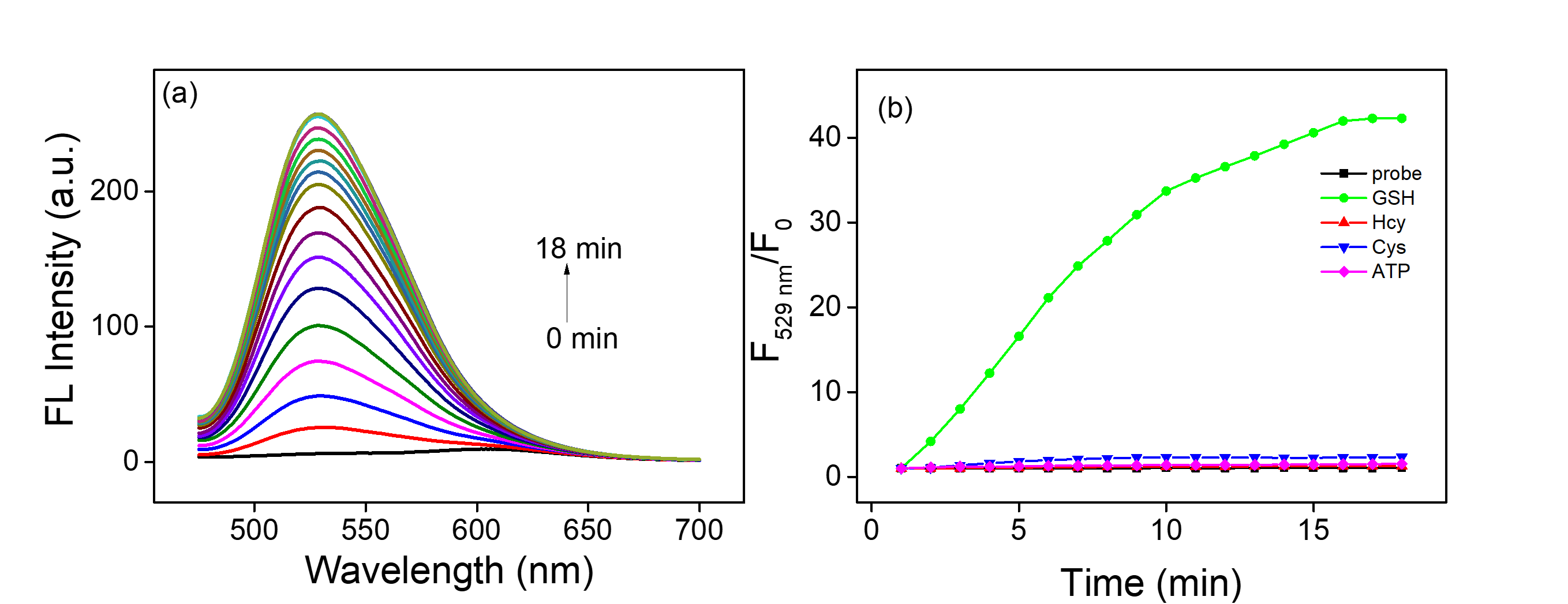


Figure S8. (a) Time-dependent fluorescence spectra of probe BCR (10 μM) upon the addition of GSH (100 μM) in DMSO/PBS (10 mM, pH = 7.4, v/v, 5/5). (b) the corresponding time-dependent fluorescence intensity changes at 529 nm. λ_ex_ = 455 nm, slit(nm): 2.5/5.


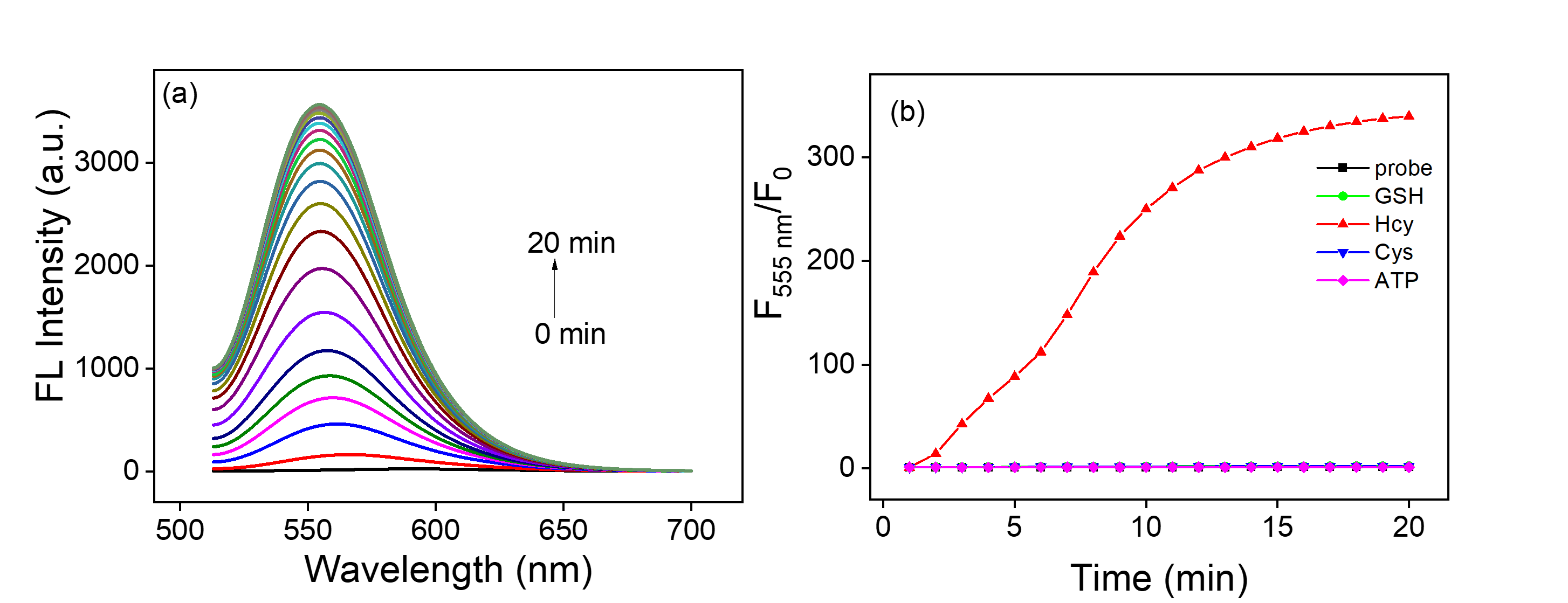


Figure S9. (a) Time-dependent fluorescence spectra of probe BCR (10 μM) upon the addition of Hcy (100 μM) in DMSO/PBS (10 mM, pH = 7.4, v/v, 7/3). (b) the corresponding time-dependent fluorescence intensity changes at 555 nm. λ_ex_ = 493 nm, slit(nm): 2.5/5.


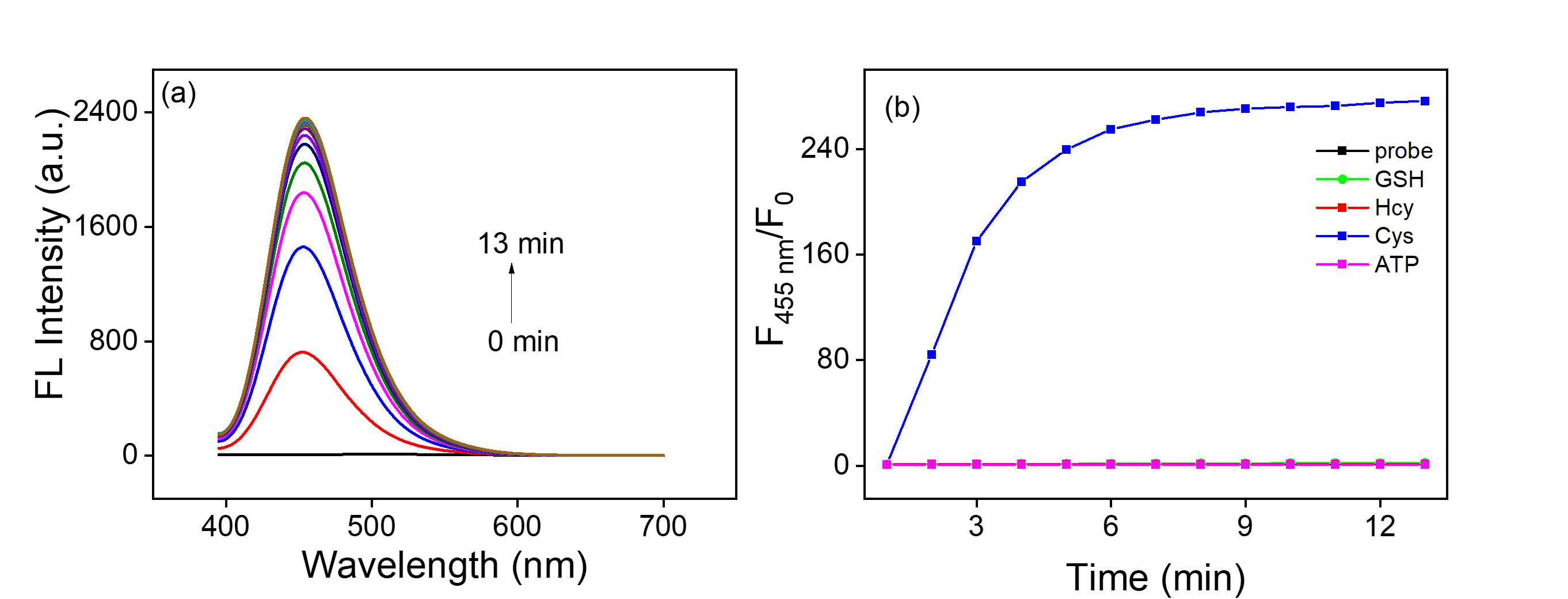


Figure S10. (a) Time-dependent fluorescence spectra of probe BCR (10 μM) upon the addition of Cys (100 μM) in DMSO/PBS (10 mM, pH = 7.4, v/v, 7/3). (b) the corresponding time-dependent fluorescence intensity changes at 456 nm. λ_ex_ = 375 nm, slit(nm): 2.5/2.5.


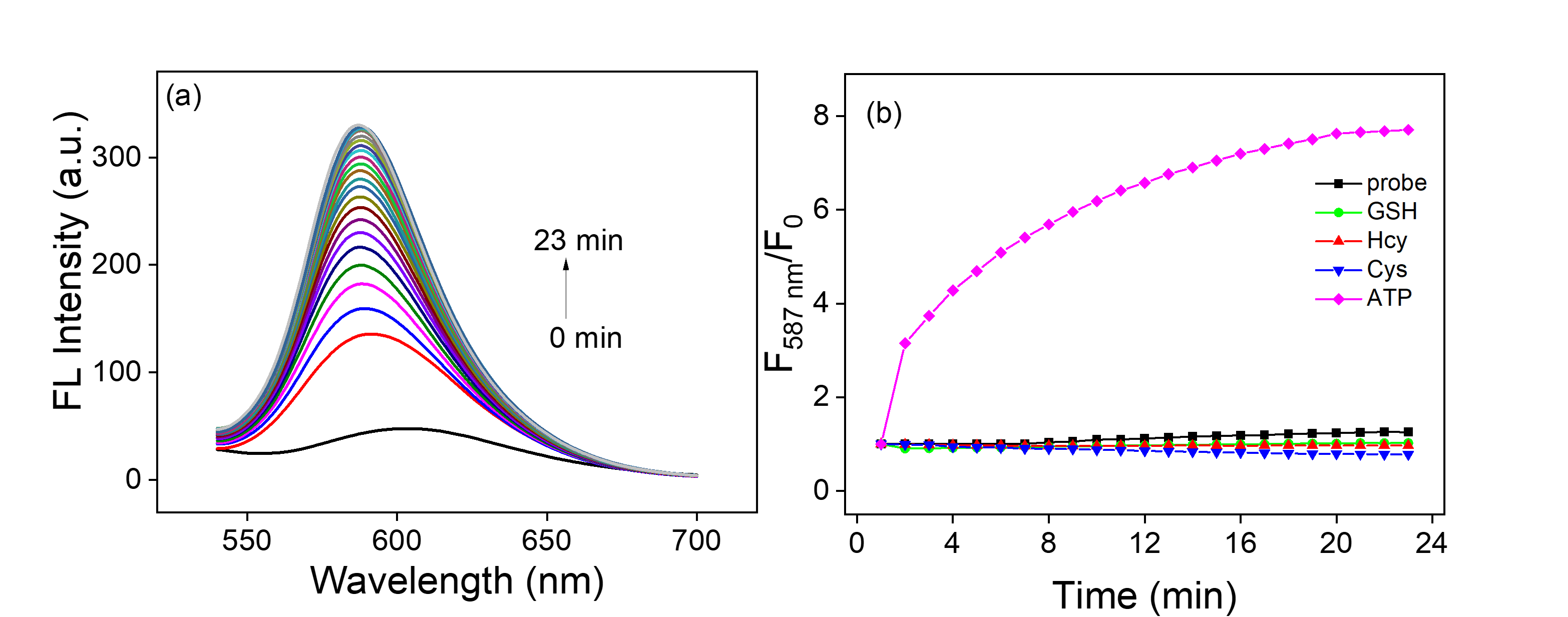


Figure S11. (a) Time-dependent fluorescence spectra of probe BCR (10 μM) upon the addition of ATP (10 mM) in MeOH/ PBS (10 mM, pH = 7.4, v/v, 5/5). (b) the corresponding time-dependent fluorescence intensity changes at 587 nm. λ_ex_ = 520 nm, slit(nm): 5/5.


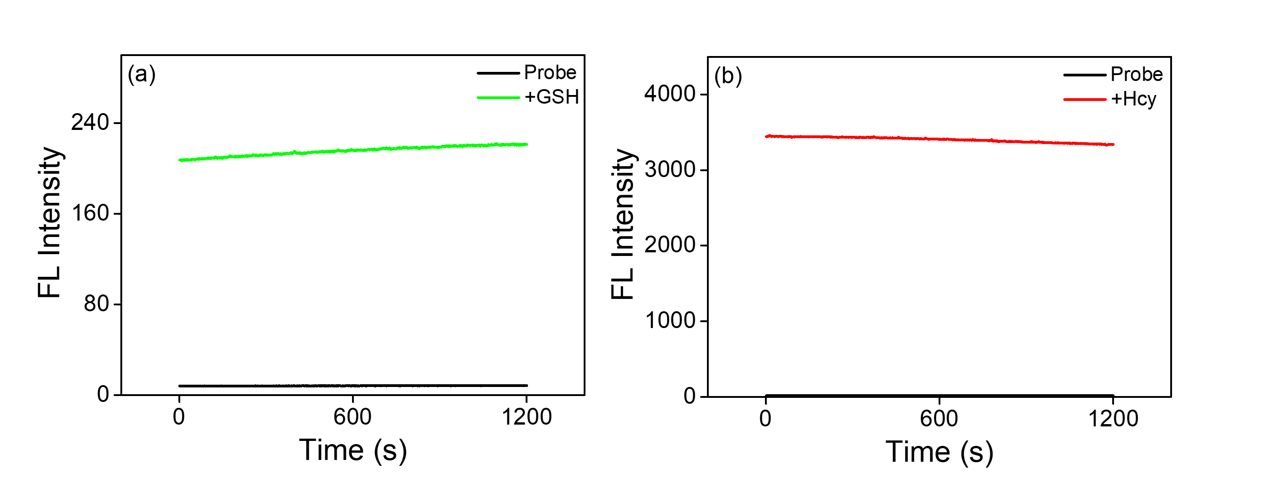


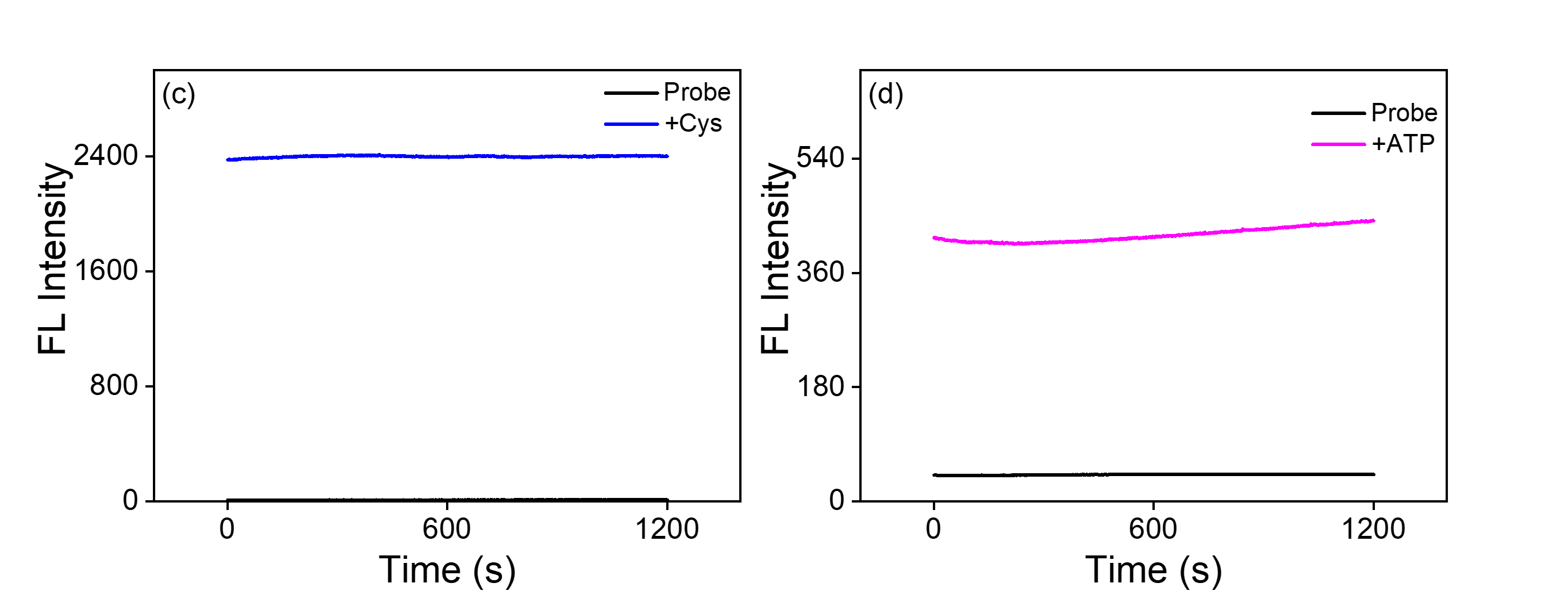


Figure S12. (a) Time-dependent fluorescence intensity changes at 529 nm for probe BCR (10.0 μM) upon addition of GSH (100 μM) for 30 min, *λ*_ex_ = 455 nm, slit (nm): 2.5/5. (b) Time-dependent normalized fluorescence intensity changes at 555 nm for probe BCR (10.0 μM) upon addition of Hcy (100 μM) for 30 min, *λ*_ex_ = 493 nm, slit (nm): 2.5/5. (c) Time-dependent normalized fluorescence intensity changes at 456 nm for probe BCR (10.0 μM) upon addition of. Cys (100 μM) for 30 min, *λ*_ex_ = 375 nm, slit (nm): 2.5/2.5. (d) Time-dependent normalized fluorescence intensity changes at 587 nm for probe BCR (10.0 μM) upon addition of. ATP (10 mM) for 30 min, *λ*_ex_ = 520 nm, slit (nm): 5/5.


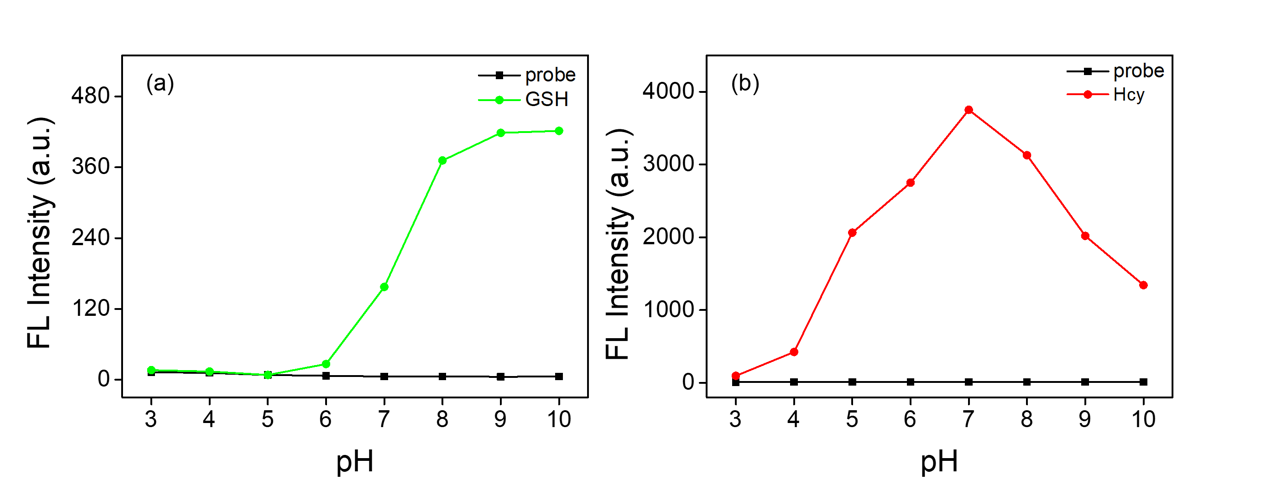


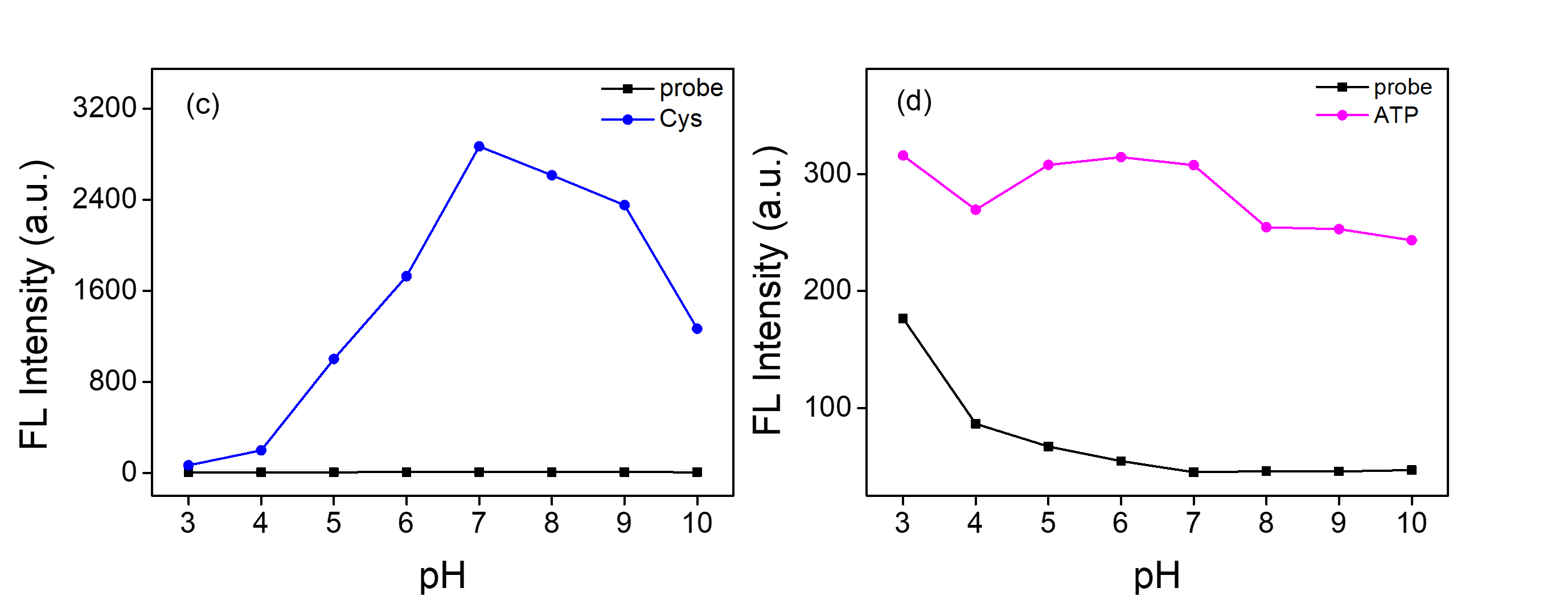


Figure S13. (a) pH-dependence of probe BCR (10 μM) towards GSH in DMSO/PBS (10 mM, pH = 7.4, v/v, 5/5) solution at 529 nm. (b) pH-dependence of probe BCR (10 μM) towards Hcy in DMSO/PBS (10 mM, pH = 7.4, v/v, 7/3). solution at 555 nm. (c) pH-dependence of probe BCR (10 μM) towards Hcy in DMSO/PBS (10 mM, pH = 7.4, v/v, 7/3) solution at 456 nm. (d) pH-dependence of probe BCR (10 μM) towards Cys in MeOH/ PBS (10 mM, pH = 7.4, v/v, 5/5) solution at 587 nm.


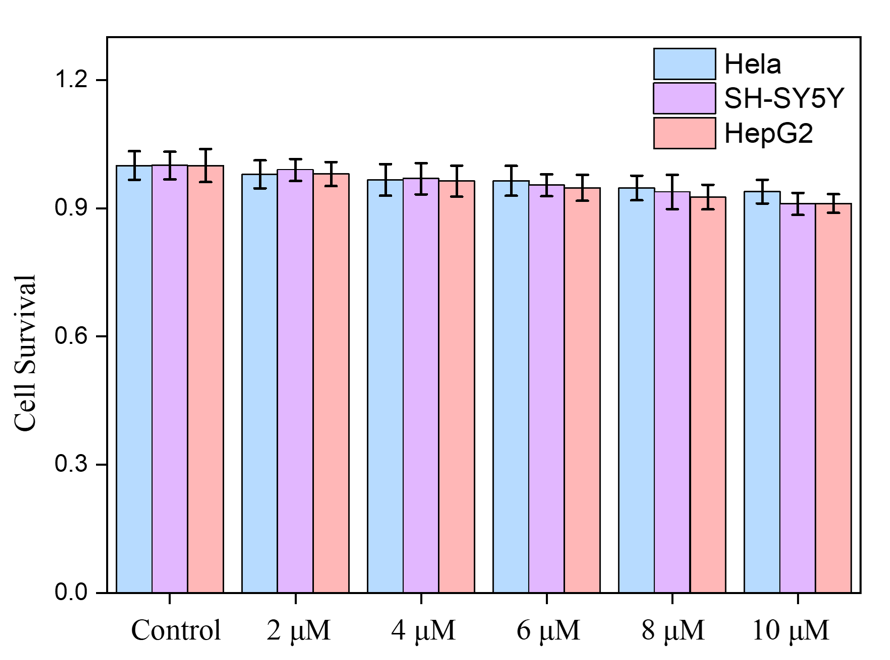


Figure S14. MTT assay for the survival rate of living HepG2, SH-SY5Y, and HepG2 cells treated with various concentrations of probe BCR for 24 h, respectively.


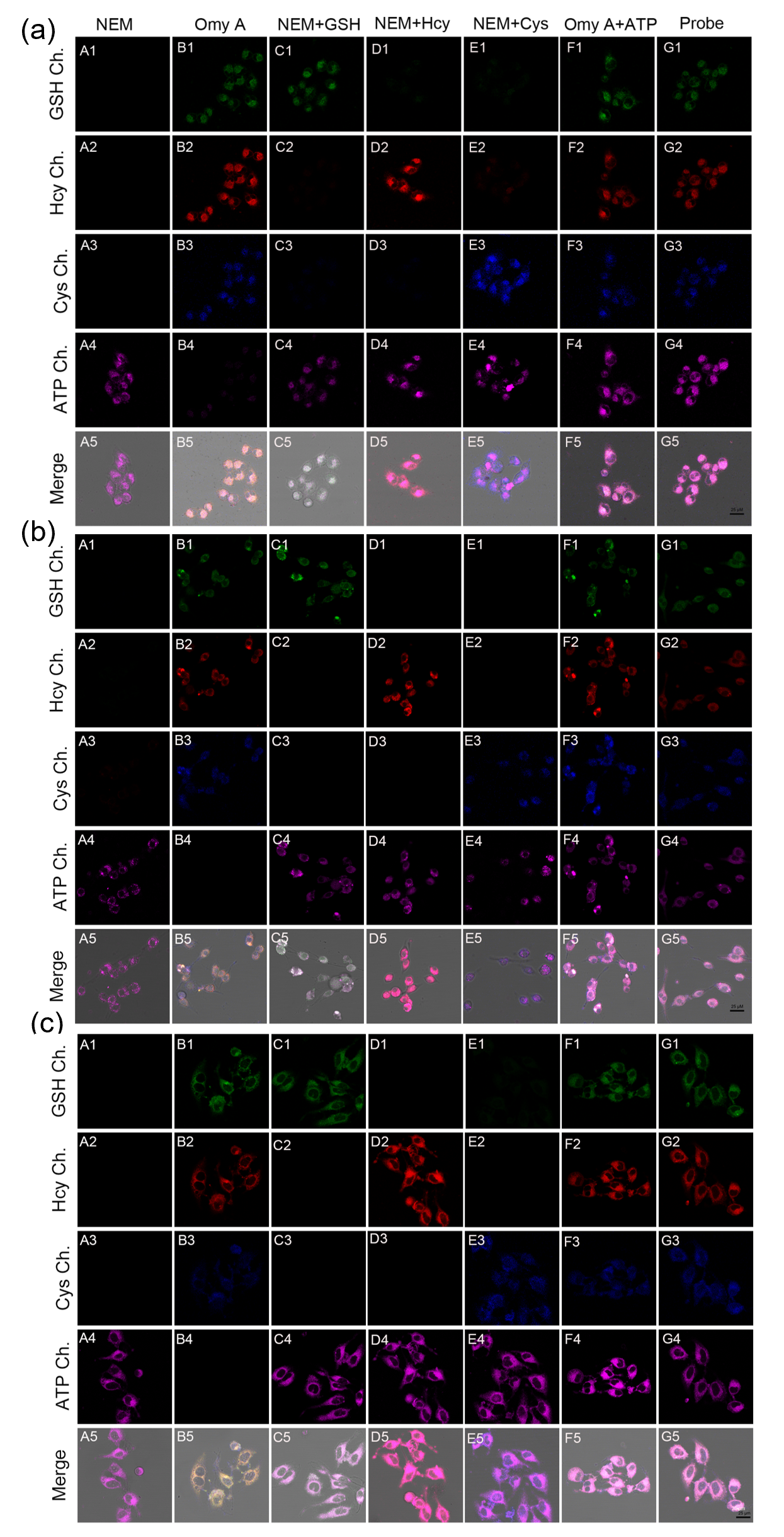


**Figure S15.** Confocal fluorescence imaging of GSH, Hcy, Cys, and ATP in (a) Hela cells, (b) SH-SY5Y and (c) HepG2 cells. The cells were pretreated with NEM (0.1 mM) for 30 min and then incubated with **BCR** (5 μM) for 45 min (A1-A4). The cells were pretreated with Omy A (50 μM) for 1 hour, and then the **BCR** (5 μM) was incubated with the cells for 45 minutes (B1-B5), and then exogenous ATP (10 mM) was added and incubated with cells for 1 hour (F1-F5). The cells were pretreated with NEM (0.1 mM) for 30 min, and then incubated with GSH/Hcy/Cys (100 μM, 30 min), respectively, and then incubated with **BCR** (5 μM) for 30 min (C1-E4). The cells were incubated with **BCR** (5 μM) for 45 min for imaging (G1-G4). λex = 405 nm, λem = 420 – 470 nm for the blue channel, λex = 458 nm, λem = 500 – 560 nm for the green channel, and λex = 488 nm, λem = 540 – 630 nm for the red channel, and λex = 514 nm, λem = 580 – 650 nm for the pink channel. Scale: 25 μm.


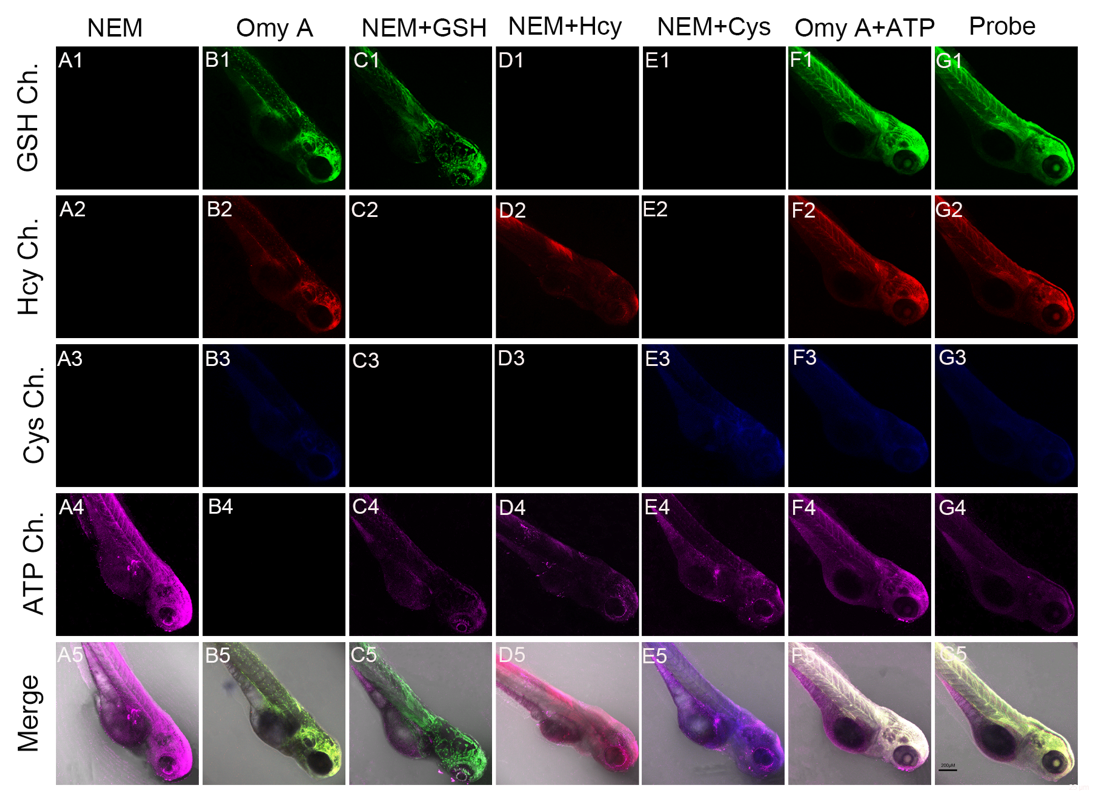


Figure S16. Confocal fluorescence imaging of endogenous GSH, Hcy, Cys and ATP in zebrafish. (A1-A5) The zebrafish were pretreated with NEM (0.1 mM) for 30 minutes, and then the probe BCR (5 μM) was incubated with the zebrafish for 45 minutes. (B1-B5) The zebrafish were pretreated with Omy A (50 μM) for 30 minutes, and then the probe BCR (5 μM) was incubated with the zebrafish for 45 minutes. (C1-E5) Pretreated zebrafish with NEM (0.1 mM) for 30 minutes, then added probe BCR (5 μM) and incubated with zebrafish for 45 minutes, then added exogenous GSH, Hcy, and Cys, respectively, and incubated with Zebrafish for 30 minutes. (F1-F5) Pretreated zebrafish with Omy A (50 μM) for 1 hour, then added probe BCR (5 μM) and incubated with zebrafish for 45 minutes, then added exogenous ATP (10 mM) and incubated with zebrafish for 1 hour. (G1-G5) Added probe BCR (5 μM) and incubated with zebrafish for 45 minutes. Scale: 200 μm.

III. ^1^H NMR, and ^13^C NMR spectra


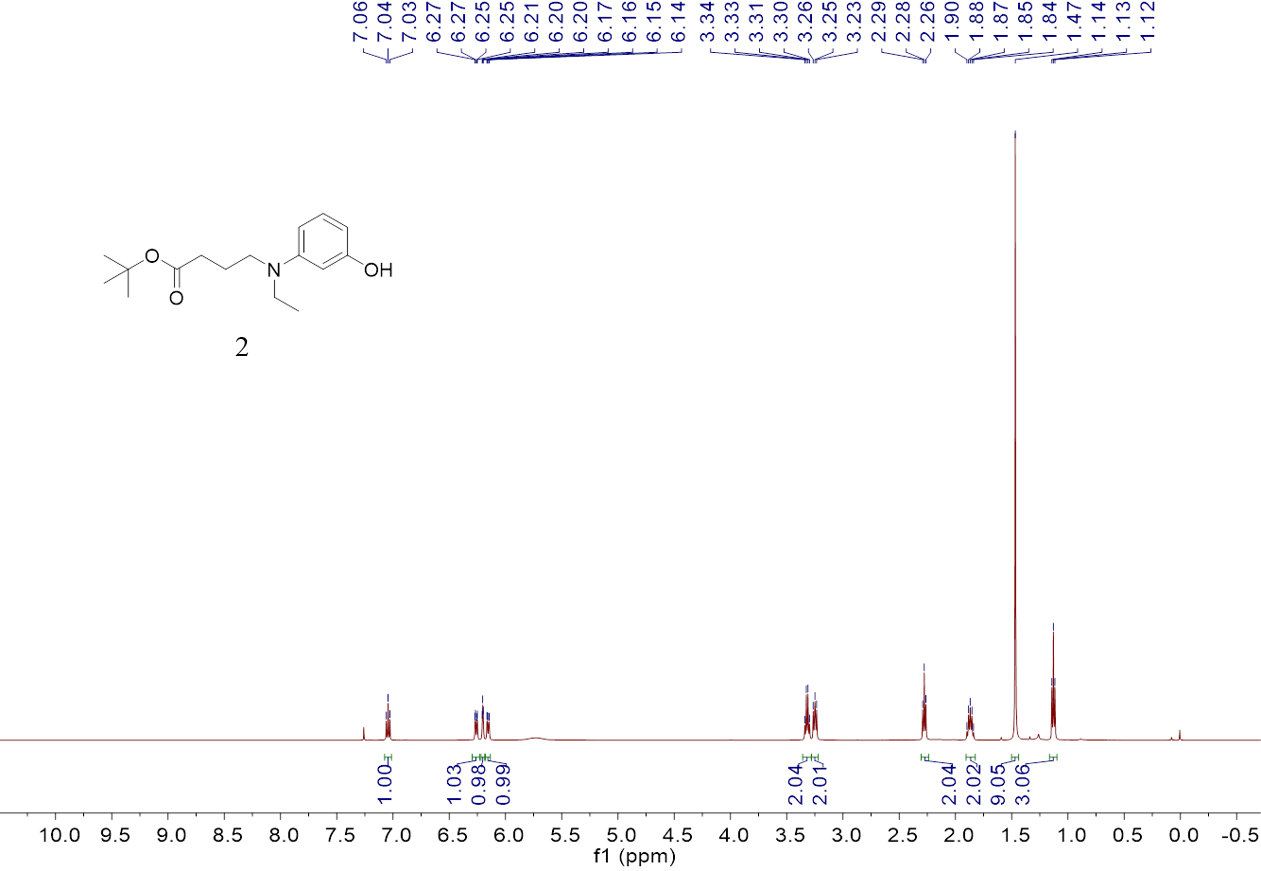
Figure S17. ^1^H NMR spectrum of compound 2 in CDCl_3_.


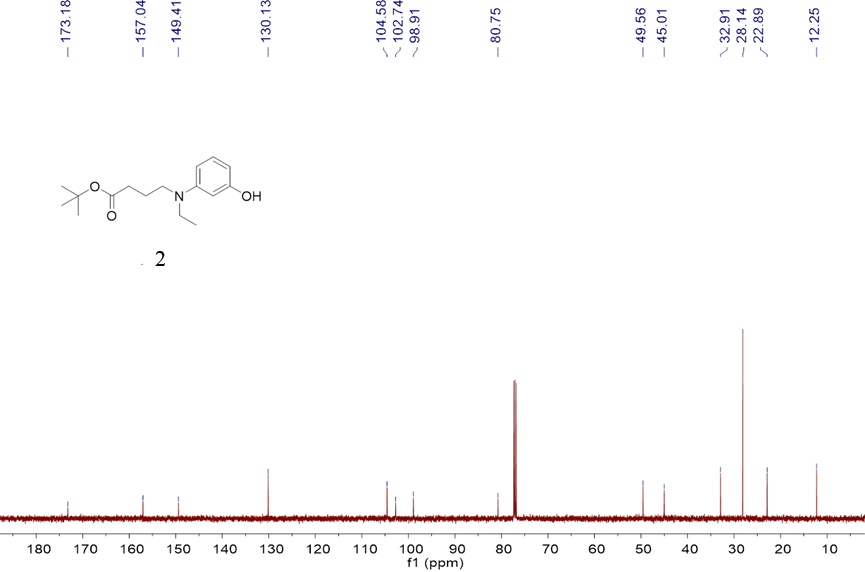


Figure S18. ^13^C NMR spectrum of compound 2 in CDCl_3_.


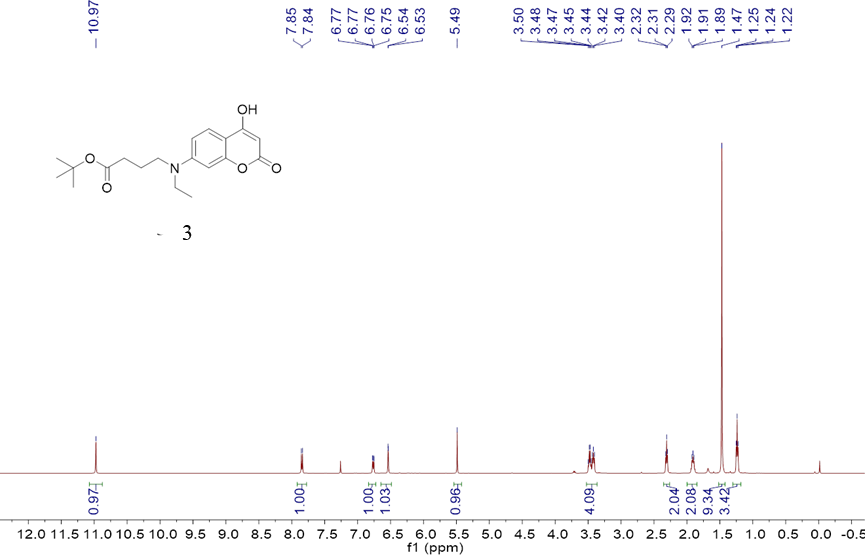
Figure S19. ^1^H NMR spectrum of compound 3 in CDCl_3_.


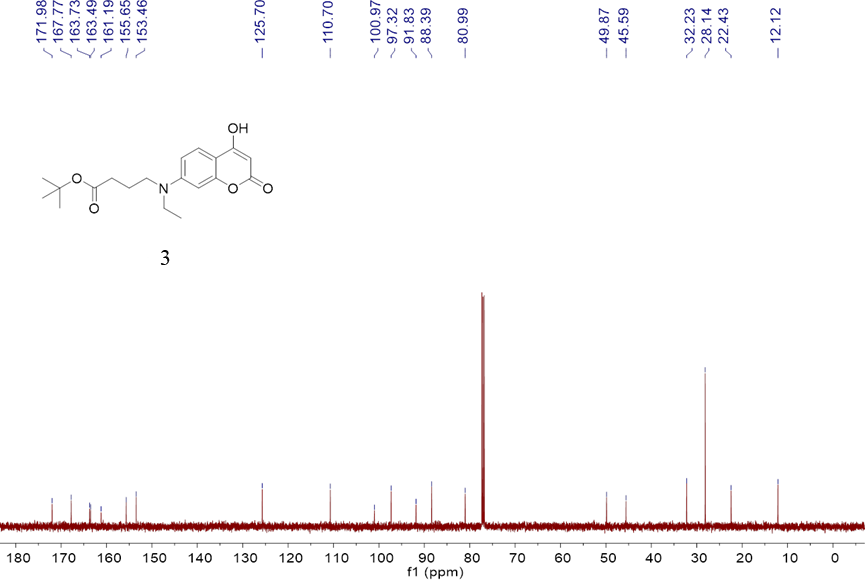
Figure S20. ^13^C NMR spectrum of compound 3 in CDCl_3_.


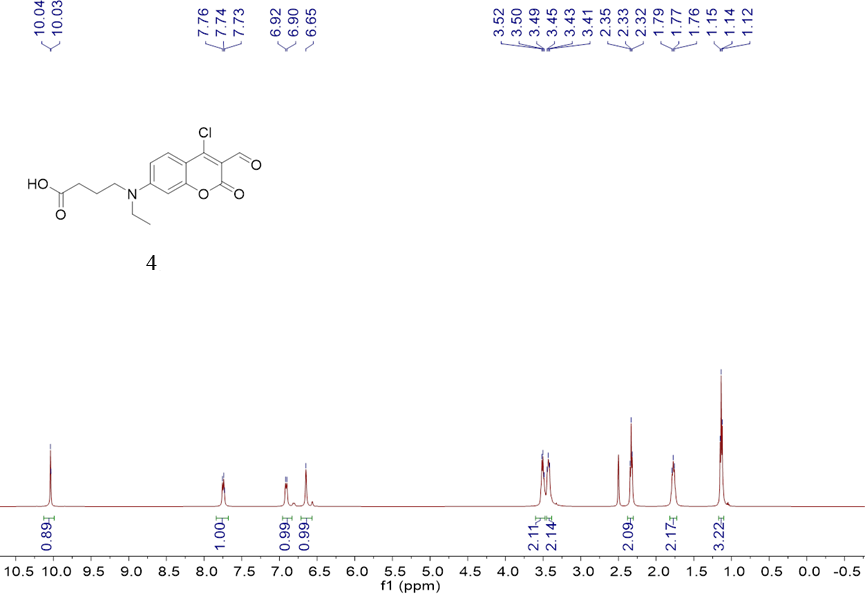
Figure S21. ^1^H NMR spectrum of compound 4 in DMSO-*d_6_*.


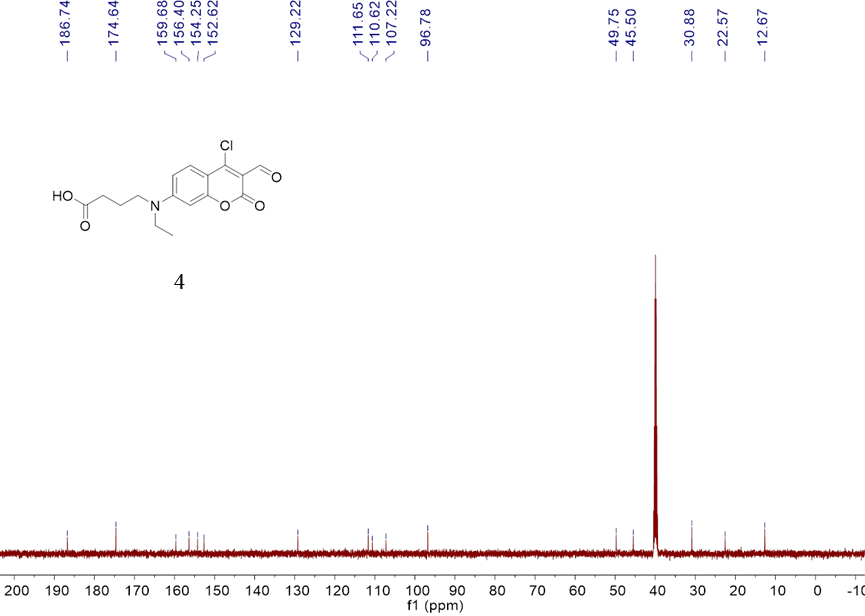
Figure S22. ^13^C NMR spectrum of compound 4 in DMSO-*d_6_*.


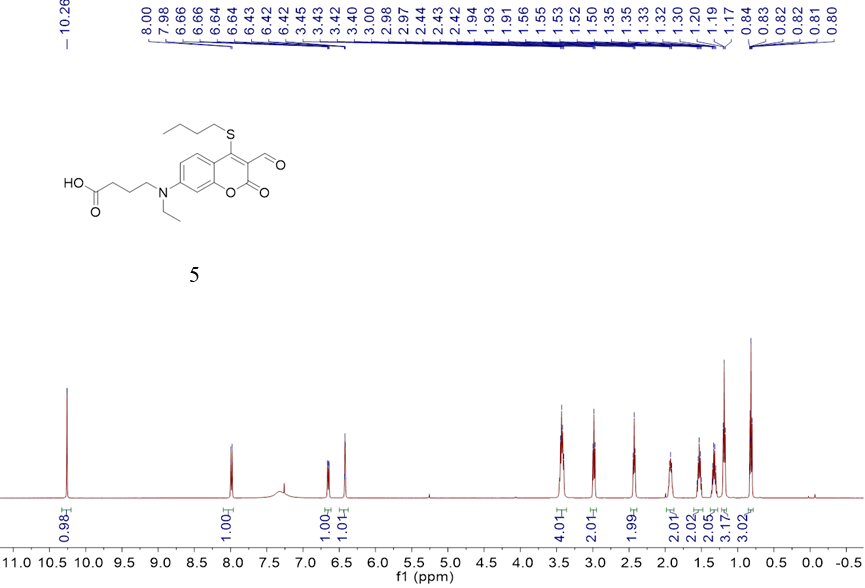


Figure S23. ^1^H NMR spectrum of compound 5 in CDCl_3_.


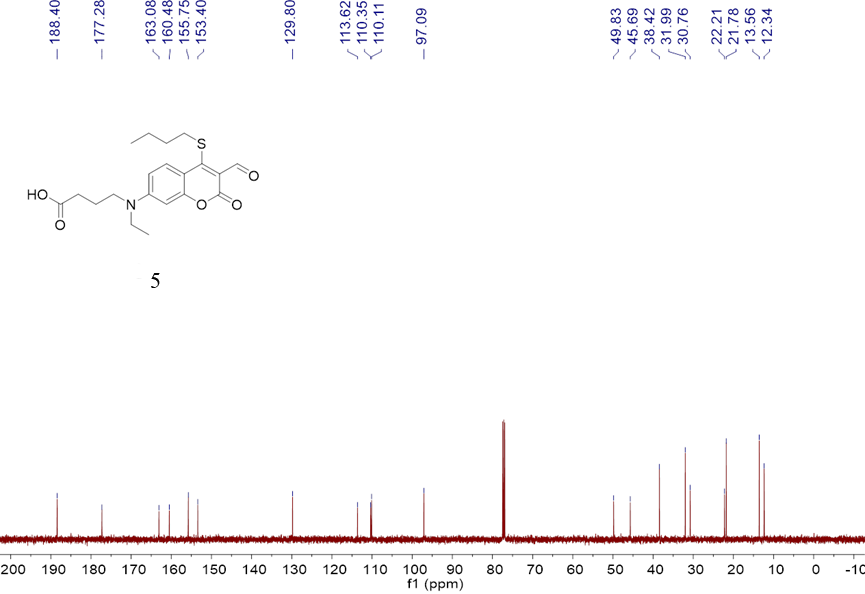


Figure S24. ^13^C NMR spectrum of compound 5 in CDCl_3_.


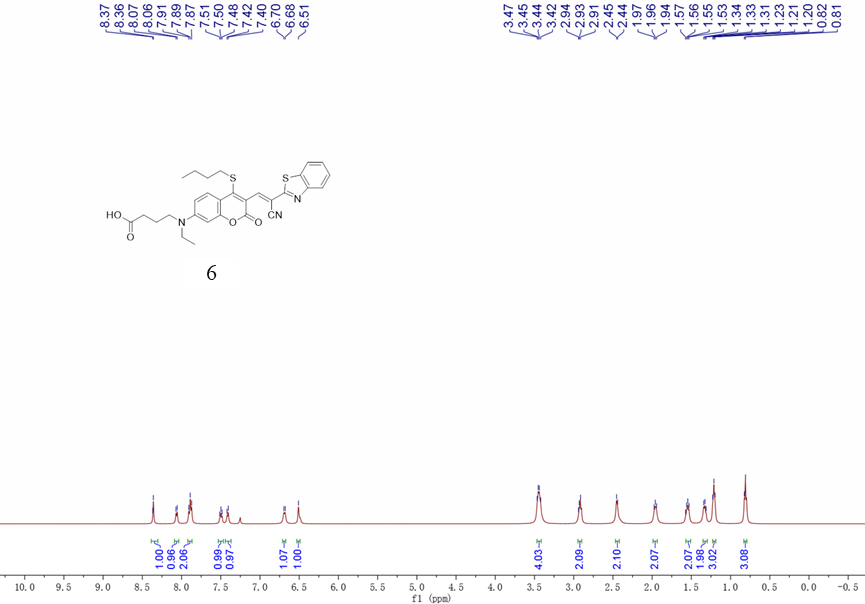
Figure S25. ^1^H NMR spectrum of compound 6 in CDCl_3_.


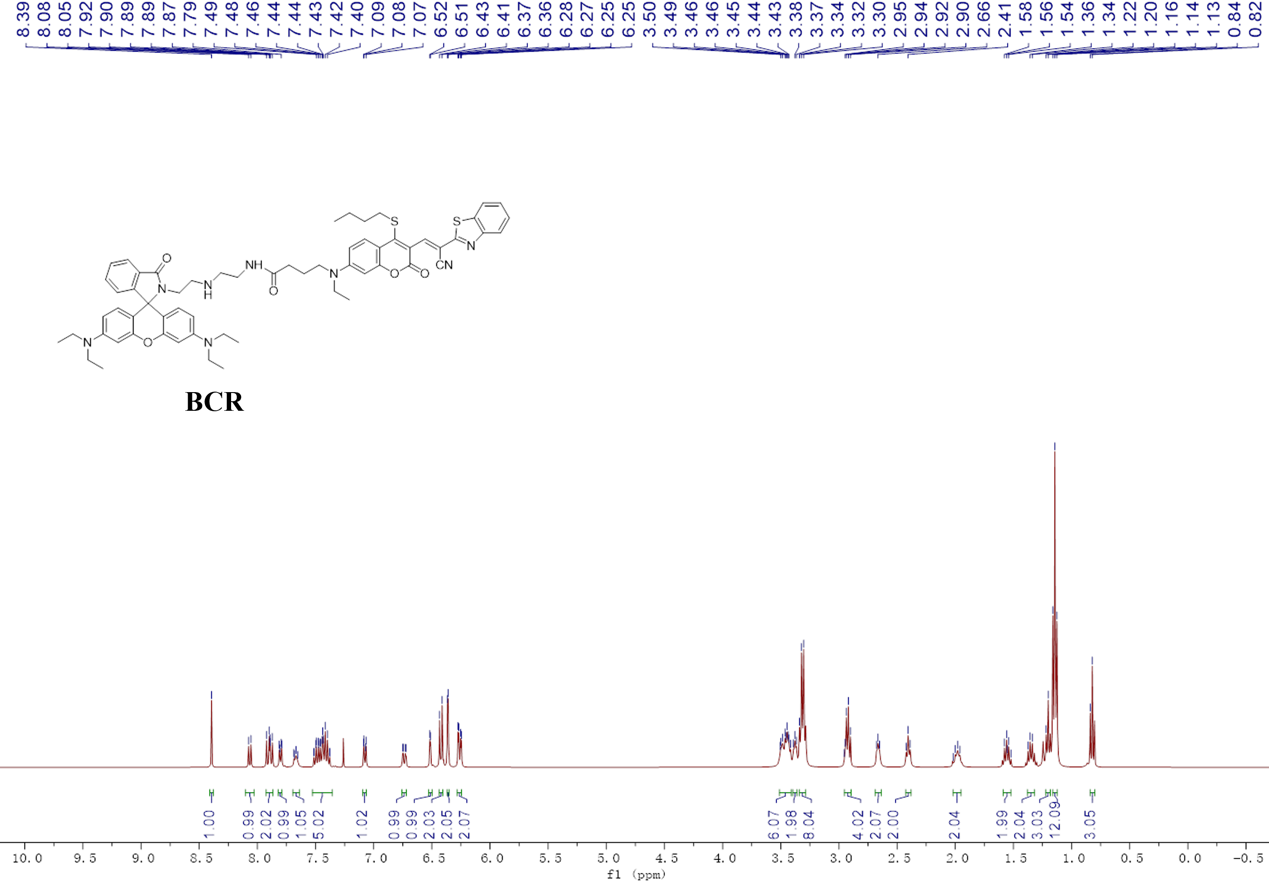


Figure S26. ^13^H NMR spectrum of BCR in CDCl_3_.


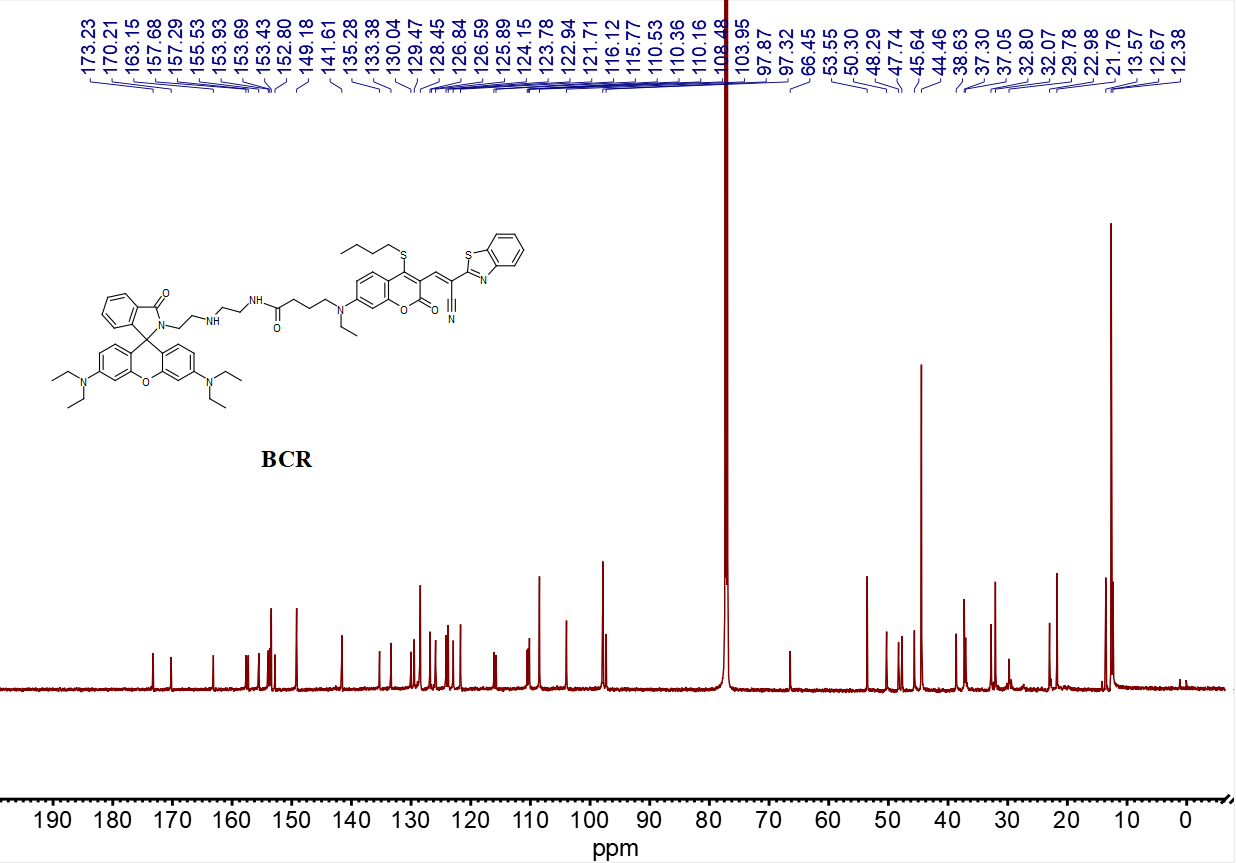


Figure S27. ^13^C NMR spectrum of BCR in CDCl_3_.


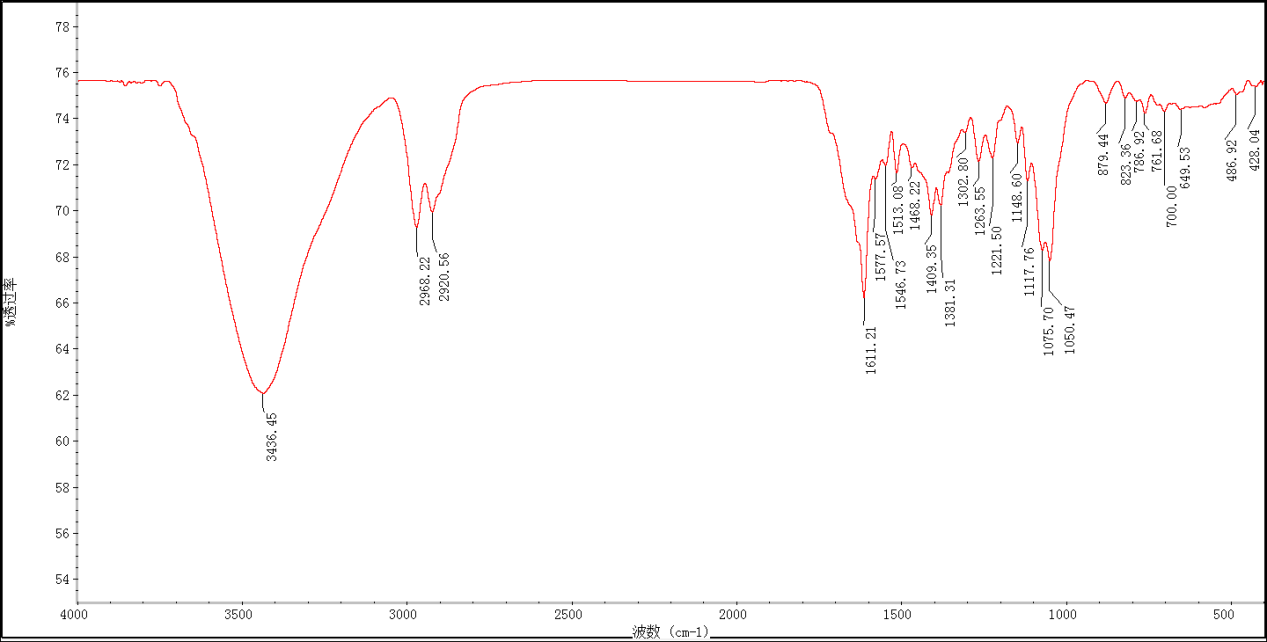


Figure S28. Infrared spectrum of BCR.


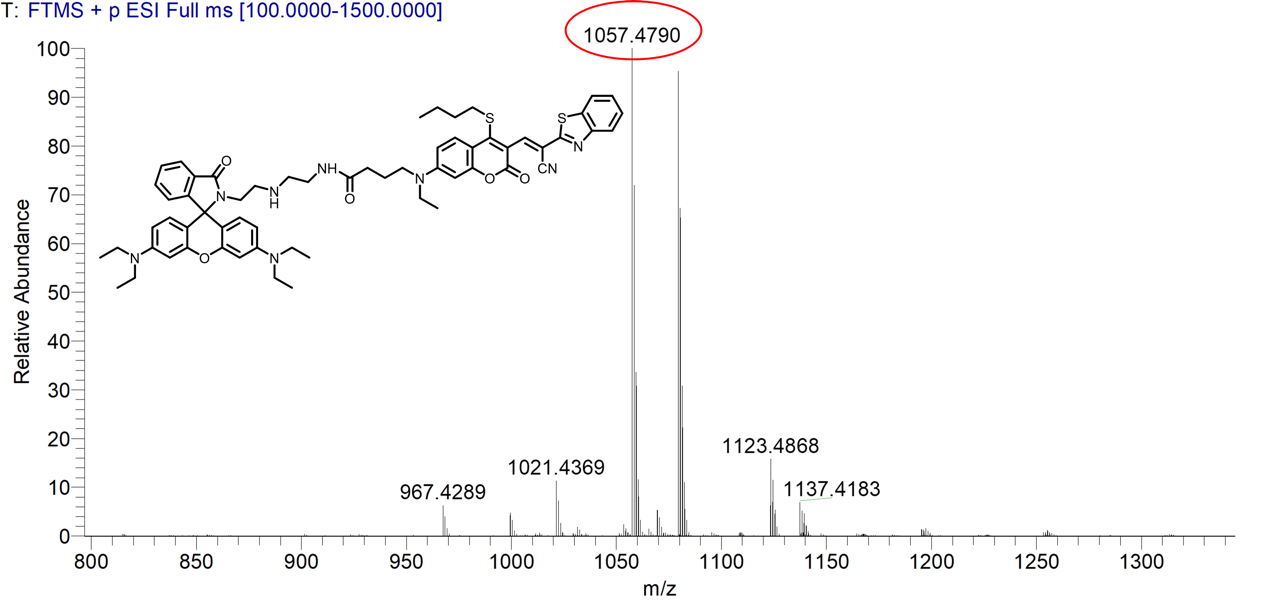


Figure S29. HRMS spectrum of probe BCR.
